# Cholesterol Corrects Altered Conformation of MHC-II Protein in *Leishmania donovani* Infected Macrophages: Implication in Therapy

**DOI:** 10.1371/journal.pntd.0004710

**Published:** 2016-05-23

**Authors:** Koushik Roy, Sapan Mandloi, Saikat Chakrabarti, Syamal Roy

**Affiliations:** 1 Infectious Diseases and Immunology Division, CSIR-Indian Institute of Chemical Biology, Kolkata, India; 2 Structural Biology and Bioinformatics Division, CSIR-Indian Institute of Chemical Biology, Kolkata, India; U.S. Food and Drug Administration and Center for Biologics Evaluation and Research, UNITED STATES

## Abstract

**Background:**

Previously we reported that Kala-azar patients show progressive decrease in serum cholesterol as a function of splenic parasite burden. Splenic macrophages (MΦ) of *Leishmania donovani* (LD) infected mice show decrease in membrane cholesterol, while LD infected macrophages (I-MΦ) show defective T cell stimulating ability that could be corrected by liposomal delivery of cholesterol. T helper cells recognize peptide antigen in the context of class II MHC molecule. It is known that the conformation of a large number of membrane proteins is dependent on membrane cholesterol. In this investigation we tried to understand the influence of decreased membrane cholesterol in I-MΦ on the conformation of MHC-II protein and peptide-MHC-II stability, and its bearing on the antigen specific T-cell activation.

**Methodology/Principal Findings:**

MΦ of CBA/j mice were infected with *Leishmania donovani* (I-MΦ). Two different anti-A^κ^ mAbs were used to monitor the status of MHC-II protein under parasitized condition. One of them (11.5–2) was conformation specific, whereas the other one (10.2.16) was not. Under parasitized condition, the binding of 11.5–2 decreased significantly with respect to the normal counterpart, whereas that of 10.2.16 remained unaltered. The binding of 11.5–2 was restored to normal upon liposomal delivery of cholesterol in I-MΦ. By molecular dynamics (MD) simulation studies we found that there was considerable conformational fluctuation in the transmembrane domain of the MHC-II protein in the presence of membrane cholesterol than in its absence, which possibly influenced the distal peptide binding groove. This was evident from the faster dissociation of the cognate peptide from peptide-MHC complex under parasitized condition, which could be corrected by liposomal delivery of cholesterol in I-MΦ.

**Conclusion:**

The decrease in membrane cholesterol in I-MΦ may lead to altered conformation of MHC II, and this may contribute to a faster dissociation of the peptide. Furthermore, liposomal delivery of cholesterol in I-MΦ restored its normal antigen presenting function. This observation brings strength to our previous observation on host directed therapeutic application of liposomal cholesterol in experimental visceral leishmaniasis.

## Author Summary

The disease visceral leishmaniasis is caused by the protozoan parasite *Leishmania donovani* (LD). One of the hallmarks of the disease is immune suppression. The parasites replicate within the macrophages and dendritic cells, and such cells are known as antigen presenting cells (APCs). APCs present peptide to T-helper cells in association with the transplantation antigen-II (MHC-II). The infected macrophages show decrease in membrane cholesterol leading to increase in membrane fluidity. The membrane cholesterol is important for maintaining conformation of membrane proteins. Here we show that conformation of MHC-II protein is altered in parasitized macrophages, which results faster dissociation of peptide from peptide-MHC-II complex as compared to normal counterpart. The conformational change in MHC-II protein is also supported by molecular dynamic simulation studies, as there is considerable structural fluctuation of MHC-II peptide binding domain in presence and absence of cholesterol. This observation indicated that cholesterol is important for maintaining conformation of MHC-II protein and stability of the peptide-MHC complex. Thus, Leishmania parasites by modulating membrane cholesterol influence above processes leading to defective T-cell stimulation in leishmaniasis. The above defects displayed by infected macrophages could be corrected by liposomal delivery of cholesterol indicating a possible therapeutic role of liposomal cholesterol in infection.

## Introduction

The disease visceral leishmaniasis or kala azar is characterized by the depression in the cellular immune response and the cause of which is largely unknown [1, 2]. The protozoan parasite, *Leishmania donovani* (LD), the causative agent of visceral leishmaniasis, replicates within the macrophage or dendritic cells of the mammalian hosts [3]. The parasites during their intracellular life cycle in the macrophages disrupt lipid rafts [4] and are unable to form synapse with antigen specific T-cells [5]. Previously, we showed that the kinetic stability of the peptide-MHC complex is compromised in LD infection [6]. The above-mentioned defects are perhaps due to increase in membrane fluidity [4], which could arise due to decrease in membrane cholesterol [5]. We also showed that splenic adherent cells of *L*. *donovani* infected hamsters show 40% decrease in membrane cholesterol and infected hamsters receiving liposomal cholesterol but not analogue liposomal cholesterol showed normal membrane cholesterol [5]. This infected hamsters receiving liposomal cholesterol but not liposomal cholesterol analogue show significant decrease in the hepatic and splenic parasite burden indicating a therapeutic role of liposomal cholesterol in experimental *L*. *donovani* infection. Similarly, ex-vivo infection of macrophages with *L*. *donovani* shows 50% decrease in membrane cholesterol [7]. Thus, infection with *L*. *donovani* leads to generalized defects in cholesterol metabolism in the infected hosts. As a part of the molecular mechanism to explain how parasite cause generalized defects, we show that miR122 maturation is compromised in *L*. *donovan*i infection. miR122 controls cholesterol metabolism in liver and its maturation from pre-miR122 to mature miR122 is controlled by the protein known as 'Dicer 1'. The parasites by using surface protease, gp63, target pre-miRNA processor Dicer 1 and thus inhibit maturation of miR122. Restoration of miR122 or Dicer 1 level in experimental visceral leishmaniasis increased serum cholesterol and reduced liver parasite burden [8]. Thus, due to absence of miR122 the overall cholesterol metabolism is decreased leading to generalized defects in cholesterol biosynthesis in *L*. *donovani* infected hosts. Furthermore, parasites may exploit membrane cholesterol for its own benefit during entry into host cells although the cause of membrane cholesterol depletion by LD parasite is not very clear yet. It is to be noted that there is an inverse correlation between membrane cholesterol and membrane fluidity [9]. The membrane cholesterol is important for the lateral mobility of the membrane proteins [9]. Previously, we also showed that INF-γ receptor subunits, R1 and R2, move beyond the Förster radii in LD infected macrophages and as a consequence IFN-γ is unable to transduce the signals in the intracellular compartment despite binding to one of its receptor subunits [7]. The above defective attributes in LD infected macrophages could be corrected by the liposomal delivery of cholesterol [7]. We have shown that there is a significant increase in lateral mobility of membrane protein in LD infected cells, which is corrected by liposomal cholesterol treatment [10].

Using conformation specific mAb directed against MHC-II protein, we could show that binding of such antibody is significantly decreased in methyl β-cyclo dextrin treated macrophages whereas the binding of non-conformation specific antibody remains unaltered [11]. CRAC (cholesterol recognition amino acid consensus) region has first been identified as a high-affinity cholesterol binding motif in the C-terminus of the peripheral benzodiazepine receptor [12]. This motif is found in many cholesterol-dependent G protein coupled receptors (GPCRs) such as receptors for oxytocin and 5HT1A, the sigma-1 receptors [13], etc. One of the major proteins of peripheral myelin, the P0 protein undergoes a cholesterol-dependent conformation change [14]. This protein has a single transmembrane domain and possesses CRAC motifs on both sides of the transmembrane segment [14]. Presence of another type of cholesterol binding motif ‘Cholesterol Consensus Motif’ (CCM) has been described in the β2-adrenergic receptor [15].

Oxytocin, cholecystokinin, galanin and nicotinic acetylcholine receptors have been shown to require membrane cholesterol for their function [16]. Many virus proteins have cholesterol binding motif. HIV gp41, influenza A M2 and SFV E1 proteins are vastly different in structure and function. However, they have one common flexible domain, which undergo conformational transition during the membrane restructuring processes and contain cholesterol binding sites [17]. There are reports of pathological effects of reduced cholesterol level on the mitochondrial dysfunction for both complexes III and IV [18].

Our earlier study showed that cholesterol interacts with transmembrane (TM) domain of MHC-II protein through CRAC motifs and alters its alpha-helical content [11]. Mutation of critical amino acids of CRAC motif (F240, L243 and F246) in TM domain weakens interaction with cholesterol. The K_d_ values observed upon interaction between cholesterol and wild type TM-domain and its corresponding mutated TM-domain were 47 nM and 2770 nM, respectively [11]. Furthermore, transfection of CHO-cells with a mutated form of Ak^β^ at the CRAC motif and wild type Ak^α^ showed normal assembly of Ak^α^ and Ak^β^ chains but such APCs were unable to stimulate T-cell as compared to transfection with wild type form [11]. The above observations clearly indicated that conformation of transmembrane MHC-II protein is governed by the binding of membrane cholesterol through its CRAC motif. Thus, altered conformation of MHC-II protein in the absence of appropriate interaction with membrane cholesterol led to concomitant decrease in the affinity of cognate peptide towards MHC-II protein and resulting in defective T-cell stimulating ability [11].

Since LD infected macrophages show decrease in membrane cholesterol, we endeavored of studying the kinetics parameters of peptide-MHC interaction in normal, LD-infected and liposomal cholesterol/cholesterol analogue treated LD-infected host cell. To gain further insight how membrane cholesterol may influence conformation of MHC-II protein, we performed all atom molecular dynamics (MD) simulation (1 μs each) with cholesterol bound (277314 atoms) and free MHC-II (330636 atoms) proteins embedded in 1-palmitoyl-2-oleoyl-sn-glycero-3-phosphocholine (POPC) bilayer. We used POPC membrane system in order to mimic overall physiological setup [19]. Structural adaptations and flexibilities within the peptide binding domain (PBD), middle domain and the transmembrane (TM) region were compared with respect to presence and absence of bound cholesterol. Our results suggest that the binding of cholesterol at the TM domain alters the inherent flexibility of the MHC-II structure and probably helps in maintaining the appropriate volume and distance of the peptidyl antigen binding pocket. Our findings extrapolated from the MD simulation data indicate that LD proteins may manipulate T-cell stimulating ability of the infected macrophages by altering conformation of MHC-II protein. To our knowledge this is the first experimental demonstration to show that intracellular *L*. *donovani* by decreasing membrane cholesterol may influence the kinetic parameters of peptide-MHC complex formation and may contribute to the defective cellular immunity in leishmaniasis and restoration of membrane cholesterol may reverse the process.

## Materials and Methods

### Ethics statement

Use of mice was approved by the Institutional Animal Ethics Committee of CSIR-Indian Institute of Chemical Biology, India. All animal experimentations were performed according to the National Regulatory Guidelines issued by CPSEA (Committee for the Purpose of Supervision of Experiments on Animals), Ministry of Environment and Forest, Govt. of India.

### Abs and other reagents

FBS, Penicillin-streptomycin, sodium bicarbonate, HEPES, 2-ME, 1,6-diphenyl-1,3,5-hexatriene (DPH), starch, cholesterol, RPMI-1640, Medium199 (M199), Giemsa and HEL (Hen Egg Lysozyme) was purchased from Sigma Aldrich (St. Louis, MO). Methanol purchased from Merck (Germany). Phosphatidylcholine (PC), 4-cholestene-3-one (cholesterol analogue), and DPPC (1,2-dipalmitoyl-*sn*-glycero-3-phosphocholine) was purchased from Avanti polar lipid Inc., (Alabaster, Alabama). ELISA assay kit for IL-2 assay was purchased from BD Bioscience (San Diego, CA). FITC conjugated mAb 11–5.2 purchased from Biolegend, San Diego. B cell hybridoma that produces mAb 10–2.16 was purchased from ATCC. m2C44 cell line, specifically recognized LACK_156-173_-major histocompatibility complex class II of H-2^d^ (A^d^) complex, was a gift from Prof. Eveylene Mougneau (Institut de Pharmacologie Moléculaire et Cellulaire, INSERM U924, Valbonne, France). Amino acid residues 156–173 (ICFSPSLEHPIVVSGSWDR) of LACK protein (defined as LACK_156–173_, Ad restricted) was synthesized using standard FMOC chemistry as described previously [6].

### T cell hybridoma

HyH12.6 (A^k^ restricted), specific for hen egg lysozyme (HEL) was gift from Prof. Peter Walden (Humbolt University, Charite’, Berlin, Germany) and LMR7.5 (A^d^ restricted), specific for 156–173 sequence of Leishmania homolog of receptors for activated C-kinase (LACK_156-173_ peptide) was gift from Prof. Eveylene Mougneau (Institut de Pharmacologie Moléculaire et Cellulaire, INSERM U924, Valbonne, France). The cell line was maintained in RPMI-1640 medium supplemented with 10% FCS and 2-ME (5 x 10^−5^ M) at 37°C with 5% CO_2_ in a humidified atmosphere.

### Monoclonal antibodies

The mAb 11–5.2 (IgG2b,k) recognize Ia.2 epitope binds close to the peptide binding groove of MHC II α chain where arginine-57 and glutamine-75 are critical for binding [20, 21]. Ia.2 is a conformational specific epitope whose expression depends on the presence of specific MHC II β chain [22] and the interaction with transmembrane domain of MHC II [23, 24]. mAb 10–2.16 recognizes β-chain of A^k^ [24].

### Animals

Balb/c mice were obtained from the animal facility of the institute and CBA/j mice were procured from National Institute of Immunology (New Delhi, India). Animals were used for experimental purposes with prior approval of the institutional animal ethics committee. Mice were housed under conventional conditions, with food and water adllibitum.

### Parasite maintenance

*Leishmania donovani (LD)* strain AG83 (MHOM/IN/1983/AG83), originally obtained from Indian kala-azar patients, was maintained in Golden Hamsters as described previously [4]. Promastigotes obtained after transforming amastigotes from spleen of infected animals were maintained in culture M199 supplemented with 10% FCS at 22°C. The culture was replenished with fresh medium every 72–96 h.

### Isolation of peritoneal exudate cells (PEC)

CBA/j and Balb/c (8–10 weeks old) mice were intraperitoneally injected with 3 ml of 4% starch. After 48 h PEC were isolated and plated on tissue culture petri dishes (1×10^6^cells/ml) or glass cover slip (1×10^5^/ml), in 10 ml or 0.5 ml respectively in complete RPMI medium for 48 h at 37°C in humified 5% CO_2_ incubator. Non-adherent cells were removed thereafter by gentle washing with serum free medium. The PECs, determined on the basis of positive staining with FITC CD11b, were ~95% CD11b^+^ [6]. For convenience, adherent PECs are defined as macrophage (MΦ) henceforth. Previous study showed that these are resting MΦ [25, 26].

### Generation of bone marrow derived of dendritic cell (DC)

Bone marrow-derived dendritic cells of Balb/c (BM-DC) were generated as described **[**27**]**. Briefly, A total of 10^6^ nonadherent bone marrow cells/ml, collected from the tibias and femurs of BALB/c mice, were seeded in a 6-well plate in the presence of rmGM-CSF (150 U/ml) and rmIL-4 (75 U/ml) and then cultured for 72 h at 37°C in humified 5% CO_2_ incubator and then supplemented with complete medium and cytokines, and subsequently cultures were fed with rmGM-CSF and rmIL-4 on days 5. After 7 days, the cells were collected. Then 2 х 10^6^ cells were transferred in a 6 well plate. The DC were used as antigen presenting cells (APCs).

### Infection of MΦ/DC with LD

Stationary phase promastigotes were used for *in vitro* infection of MΦs/DC (derived from Balb/c). The MΦs/DC after overnight incubation in complete medium, were challenged with LD promastigotes (macrophage/DC to parasite ratio 1:10) and incubated further for 6 h at 37°C. Excess parasites were then washed off with serum free medium. The MΦs/DC were then incubated further for 48 h and intracellular parasites were enumerated as described previously [28]. Briefly, at endpoints the cover slips were washed with PBS, microscopically and the results were expressed as % infected as well as the number of parasites/ 100 MΦ, dried, fixed with 100% methanol and stained with 10% Giemsa. It was observed that at 6 h average parasite/ cell was 5 and 48 h average parasite/ cell was 11 (S1 Fig). 85–90% of MΦs were infected MΦs.

### Liposome preparation and treatment

Liposomal cholesterol or liposomal cholesterol analogue were prepared either with cholesterol or cholesterol analogue (4-cholestene-3-one) and phosphatidyl-choline at a molar ratio of 1.5:1 as previously described [5]. DPPC liposome was prepared as described in [7]. To alter the fluidity of cells, 10^5^ cell /100μl were incubated with 10 μl liposomes for 20 h at 37°C. The cells were then washed three times in serum-free RPMI 1640 medium and finally resuspended in 10% FCS containing RPMI 1640.

### Measurement of fluorescence anisotropy (FA)

The membrane fluidity of cells was measured following the method described by Shinitzky *et al*. [29]. Briefly, the fluorescent probe DPH was dissolved in tetrahydrofuran at 2 mM concentration. To 10 ml of rapidly stirring PBS (pH 7.2), 2 mM DPH solution was added. For labeling, 10^6^ cells were mixed with an equal volume of DPH in PBS (C_*f*_ 1 μM) and incubated for 2 h at 37°C. Thereafter the cells were washed thrice and resuspended in PBS. The DPH probe bound to the cellular membrane was excited at 365 nm and the intensity of emission was recorded at 430 nm in a spectrofluorometer. The FA value was calculated using the equation: FA = [(I_II_−(G×I_I_)]/ [I_II_ +(2×G×I_I_)], where I_II_ and I_I_ are the fluorescent intensities oriented parallel and perpendicular to the direction of polarization of the excited light.

### Stimulation of T cell hybridoma in the context of antigen presenting cells (APCs) and peptide/protein

The ability of normal MΦ (N-MΦ), infected MΦ (I-MΦ), infected MΦ treated with liposomal cholesterol (I-MΦ-CL), infected MΦ treated with liposomal cholesterol analogue (I-MΦ-AL), infected MΦ treated with liposomal DPPC (I-MΦ-DPPC) and normal MΦ treated with liposomal cholesterol (N-MΦ-CL) were used as APCs to stimulate anti-HEL T cell hybridoma (HyH12.6) and anti-LACK T cell hybridoma (LMR 7.5) in the presence of HEL protein and LACK_156-173_ peptide respectively. Briefly, 5×10^5^ T cell hybridomas were cocultured with 1X10^5^ appropriate MΦs in the presence of respective antigen. Both T cell and MΦs were kept for 24 h in complete medium at 37°C in humified 5% CO_2_. The resulting culture supernatant was assayed for IL-2 by ELISA.

### Analysis peptide-MHC stability on cells surface using FACS

Since mAb, m2C44 recognizes LACK_156-173_-A^d^ [30] the peptide-MHC stability was studied in MΦ derived from Balb/c mice. MΦs were pulsed with 20 μM LACK_156-173_ peptide for 6 h at 37°C followed by washing and then fixed with 4% paraformaldehyde followed by washing (this is defined as ‘0’ h). We have studied the stability of the peptide-MHC complex in the peptide pulsed fixed MΦ as described by others [31, 32]. Then the cells were incubated with 50 μM OVA_323-339_ (A^d^ restricted) in order to prevent rebinding of the dissociated LACK_156-173_ peptide [33, 34]. The cells were incubated with 400 μl of m2C44 culture supernatant for 1 h followed by stained with goat anti-mouse IgG FITC as described [30]. MFI of 5000 cells were measured at 0, 2, 4, 8 and 12 h.

### Functional analysis of peptide-MHC stability

MΦs were pulsed with 20 μM LACK_156-173_ for 6 h at 37°C [34] and then fixed with 4% paraformaldehyde followed by washing (this is defined as ‘0’ h) and incubated with 50 μM OVA_323-339_. Then the cells were cocultured with T cell hybridoma (LMR7.5) at 0, 2, 4, 8, 12 and 24 h. The resulting IL-2 production in the supernatant was measured by ELISA [4].

### Cell surface expression of MHC-II

Conformation specific mAb is available for H-2^k^ haplotype (11–5.2) recognize Ia.2 epitope but not for H-2^d^, therefore conformational change of MHC-II protein was studied in MΦ derived from CBA/j mice. Ia.2 epitope expression was measured using FITC label mAb 11-.5.2. The total cell surface expressions of MHC-II were measured by anti-mouse A^k^ (10–2.16) [24]. The cells were stained with goat anti-mouse FITC in PBS containing 5% FCS. The MFI for FITC was determined in a FACS ARIA-II system (BD Bioscience, San Diego, CA).

### Molecular dynamics simulation system

Molecular dynamics (MD) simulations of the MHC-II embedded in 1-palmitoyl-2-oleoyl-sn-glycero-3-phosphocholine (POPC) membranes were carried out in the presence and absence of cholesterol using GROMACS 4.6.1 [35]. The chemical structure of POPC and cholesterol is shown in S2 Fig. In our previous study, X-ray coordinates of MHC-II protein extracellular domain structure [PDB ID: 2IAD] was attached with computational model of TM domain using Modeler v9.9 [11]. The full length MHC-II model was validated extensively and utilized in molecular docking of cholesterol to the CRAC motifs of MHC-II protein [11]. In the same study, experimental validation of the predicted binding sites further added support to the reliability of the three-dimensional (3D) model [11]. Hence, in this study, same 3D models of MHC-II with bound and unbound cholesterol [11] were utilized to understand the effect of cholesterol on peptide binding. POPC bilayer was created with 512 POPC molecules and for uniform distribution of POPC, the bilayer was equilibrated for 60 nano seconds (ns). Docked MHC-II-cholesterol was inserted into the equilibrated POPC bilayer using the GROMACS *g_membed* tool (S2 Fig) [36]. All the systems were solvated with SPC (simple point charge) waters [37] and system-neutralizing sodium ions were added. GROMOS96 53A6 force field [38] was used for all the molecules. Size, duration, and other relevant details regarding the simulation runs are provided in Table 1. Super-computing facility located at CSIR-fourth paradigm institute, Bangaluru, India, was utilized to perform 1μs-scale simulations of MHC-II-cholesterol system comprising of approximately 3 lakhs atoms.

**Table 1:** Summary of MD simulations.

	Run no.	System size (atoms)	Duration of simulation	No. of POPC molecule	No. of water bead	No. of CHL
With CHL	1	277314	1μs	489	82290	2
With CHL	2	277314	1μs	489	82290	2
With CHL	3	277314	1μs	489	82290	2
Without CHL	1	330636	1μs	490	100066	0
Without CHL	2	330636	1μs	490	100066	0
Without CHL	3	330636	1μs	490	100066	0

Details of simulations performed with and without cholesterol (CHL) systems.

### Simulation conditions

In all simulations, a leap-frog integrator [39] was used with a 2 fs time step. Periodic boundary conditions together with the usual minimum image convention were used in all three directions. To remove steric clashes and to avoid drastic rearrangement during setup, all the systems were energy-minimized using steepest descent, conjugate gradient and positional restraint methods. All covalent bonds in the system were constrained to their equilibrium values with the LINCS algorithm [40].

Cutoff radius of 12 Å was employed for van der Waals and electrostatics interactions. The particle-mesh Ewald (PME) algorithm [41] was applied to treat long-range electrostatic interactions. The update of the neighbor list for short-range non-bonded interactions was done every 10 MD steps. The temperature for each group was coupled using the Nose-Hoover thermostat algorithm [42] with a coupling constant of 2.5 ps to maintain a constant temperature of 300K during simulation. Semi-isotropic pressure was maintained using the Parrinello-Rahman algorithm [43] with a pressure of 1 bar independently in the plane of the membrane and perpendicular to the membrane, a coupling constant of 5 ps, and a compressibility of 4.5e-5. The 3 MD runs (see Table 1 for more detail) of 1μs each (with and without cholesterol system) were taken for calculating the averages. The atomic coordinates were saved at every 2 ps for the analyses. GROMACS tools were used to do analyses of the trajectories generated from the MD simulations. A cholesterol molecule was defined to be bound to a particular transmembrane helix or amino acid residue (site) of MHC-II if it was within 0.5 nm of the previously described binding sites [11].

The calculations of *root mean square deviation* (RMSD) and *root mean square fluctuation* (RMSF) were made with respect to the starting structure. The time dependent change of the solvation area was calculated using *g_sas* whereas the helicity was estimated by the *g_helix* module of GROMACS. Tilt and twist of the helices were calculated using the *g_helixorient*. ProDy [44] was used to calculate the all-to-all cross-correlation of fluctuation within the MHC-II molecule. Mean correlation was calculated by averaging the correlation of fluctuation between any two residues from the three independent simulation runs.

## Results

### Restoration of membrane fluidity of infected macrophages by liposomal forms of cholesterol or DPPC but not by cholesterol analogue

**Fig 1:**
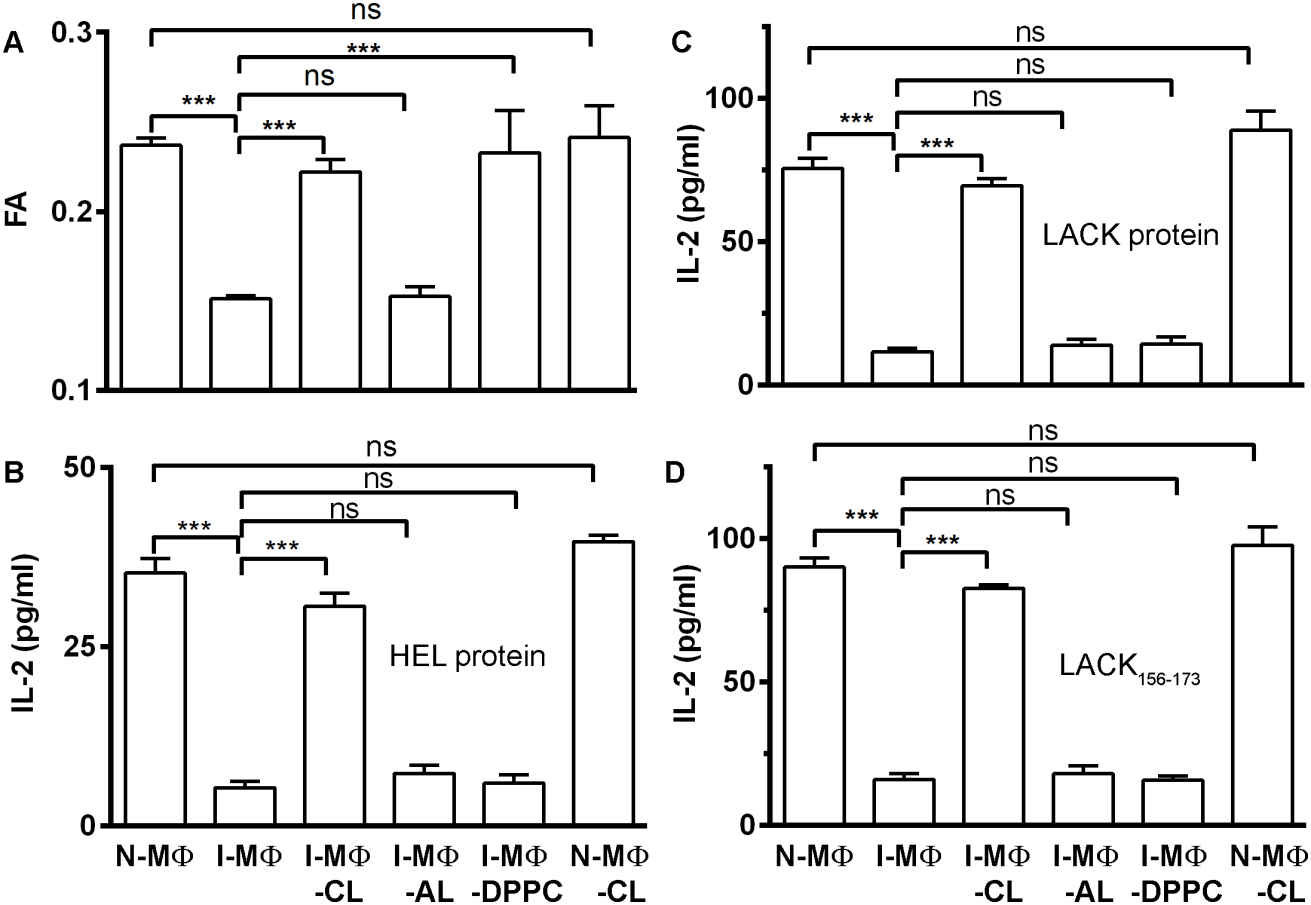
Determination of membrane fluidity in terms of Fluorescence Anisotropy (FA) and T cell stimulating ability of normal, infected and liposome treated infected MΦ. **A,** The FA (Fluorescence Anisotropy) of normal MΦ (N-MΦ), infected MΦ (I-MΦ), infected MΦ treated with liposomal cholesterol (I-MΦ-CL), infected MΦ treated with liposomal cholesterol analogue (I-MΦ-AL), infected MΦ treated with liposomal DPPC (I-MΦ-DPPC) and normal MΦ treated with liposomal cholesterol (N-MΦ-CL) was measured using DPH as a probe. The fluorophore was excited at 365 nm, emission intensity was recorded at 430 nm and FA was calculated as described in the materials and methods. **B,** anti-HEL T cell hybridoma (HyH12.6, A^k^ restricted) were cocultured with N-MΦ, I-MΦ, I-MΦ-CL, I-MΦ-AL, I-MΦ-DPPC and N-MΦ-CL in presence of 15 μM HEL protein. For stimulation of HyH12.6, MΦs were derived from CBA/j mice. **C,** anti-LACK T cell hybridoma (LMR7.5, A^d^ restricted) were cocultured with N-MΦ, I-MΦ, I-MΦ-CL, I-MΦ-AL, I-MΦ-DPPC and N-MΦ-CL in presence of 5 μM LACK protein. For stimulation of LMR7.5, MΦs were derived from Balb/c mice. **D,** anti-LACK T cell hybridoma (LMR7.5) were cocultured with N-MΦ, I-MΦ, I-MΦ-CL, I-MΦ-AL, I-MΦ-DPPC and N-MΦ-CL in presence of 5 μM LACK_156-173_ peptide. The resulting IL-2 production in the culture supernatant was assayed by ELISA as a read out of T-cell stimulation.

The MΦ from CBA/j mice were infected with LD (I-MΦ) and treated with liposomal cholesterol (I-MΦ-CL) or liposomal cholesterol analogue (I-MΦ-AL) or liposomal DPPC (I-MΦ-DPPC). Similarly, normal MΦ (N-MΦ) were also treated with liposomal cholesterol (N-MΦ-CL). The membrane fluidity of N-MΦ, I-MΦ, I-MΦ-CL, I-MΦ-AL, I-MΦ-DPPC and N-MΦ-CL was measured in terms of fluorescence anisotropy (FA) using DPH as a fluorescence probe. It was observed that there was significant decreased in FA in I-MΦ which was restored to normal in I-MΦ-CL and 1-MΦ-DPPC but not in I-MΦ-AL. Interestingly in N-MΦ-CL insignificant change in FA values compared to untreated control MΦ (Fig 1A).

### Only liposomal cholesterol treatment restored T-cell stimulating ability of infected macrophages/ dendritic cells

MΦ/DC from Balb/c (H-2^d^) and CBA/j (H-2^k^) were used as antigen presenting cells (APCs) to stimulate respective MHC-II restricted T-cell hybridomas, HyH12.6 (A^k^ restricted) and LMR7.5 (A^d^ restricted). The IL-2 production from T-cell hybridomas in the presence of appropriate APCs and antigens was used as a functional read out of T-cell activation. It was observed that T-cell stimulating ability of I-MΦ of Balb/c or CBA/j origin was decreased by ≈ 6 to 7 fold as compared to N-MΦ (Fig 1B and 1C). Furthermore, only liposomal cholesterol treatment of I-MΦ restored the APC function to normal regardless of the source of MΦ. On the other hand, cholesterol analogue liposome or DPPC liposome treatment of I-MΦ failed to show any further improvement in their ability to stimulate T-cell. Interestingly enough, DPPC liposome treatment of I-MΦ restored the membrane fluidity but not their APC function (Fig 1B and 1C). It was observed that LACK protein and LACK peptide (LACK_156-173_) showed similar results in terms of T cell response (Fig 1D).

Similarly, I-DC were unable to stimulate T cell hybridoma (LMR7.5). Only, liposomal cholesterol treatment of I-DC (I-DC-CL) can restore APC function not by others treatment such as liposomal cholesterol analogue or liposomal DPPC (S3 Fig).

### Decreased binding of conformation (11–5.2) but non-conformation (10.2.16) specific anti A^k^ mAb to I-MΦ corrected by liposomal cholesterol treatment

**Fig 2:**
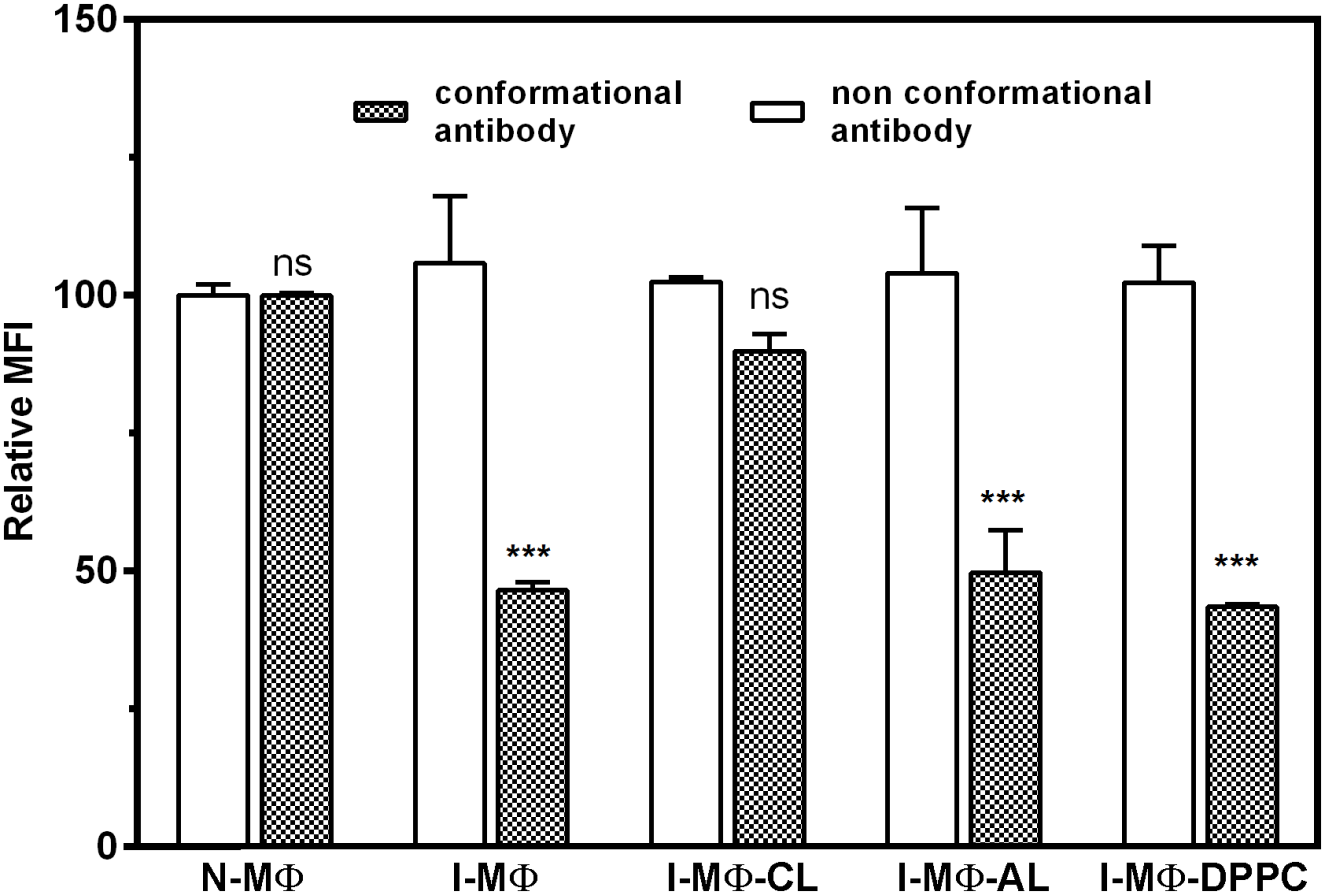
LD infection show reduced binding of conformational antibody but not non-conformational antibody. The binding of mAb 11–5.2 (conformational antibody) to N-MΦ, I-MΦ, I-MΦ-CL, I-MΦ-AL and I-MΦ-DPPC was determined to assess cell surface expression of Ia.2 epitope (filled bar). The cells were stained with FITC conjugate mAb 11–5.2. Similarly binding of mAb 10–2.16 (non-conformational antibody) to N-MΦ, I-MΦ, I-MΦ-CL, I-MΦ-AL and I-MΦ-DPPC was determined to assess total expression of A^k^ (blank bar). The cells were stained with mAb 10–2.16 followed by FITC conjugated goat anti mouse IgG. The binding was expressed in terms of MFI. *** represents p<0.0005, ns represent non-significant.

Because of the availability of the conformation specific anti A^k^ mAb (11–5.2) such studies were carried out with MΦ of CBA/j origin. The binding ability of two different anti A^k^ mAb was studied in N-MΦ, I-MΦ, I-MΦ-CL. The binding was monitored in terms of fluorescence intensity and results were expressed as MFI. It was observed that the binding of 11–5.2 was decreased by about 60% in I-MΦ as compared to N-MΦ whereas binding of non-conformation specific antibody (10–2.16) remain unaltered. Similar trend was maintained in I-MΦ-AL and I-MΦ-DPPC. However the binding of 11–5.2 was restored to normal in I-MΦ-CL (Fig 2).

### Altered stability of MHC-II in absence of bound cholesterol

Molecular dynamic simulation was carried out using a three-dimensional (3D) model of full length MHC-II dimer structure [11] docked with cholesterol. Structural stability of the MHC-II in presence and absence of cholesterol was investigated based on properties such as root mean square deviation (RMSD) and root mean square fluctuation (RMSF) of the full length MHC-II and the bound peptide. The RMSD is the average displacement of the atoms at an instant of the simulation relative to the first frame of the simulation. The RMSD of MHC-II in presence of cholesterol was increased from ~1Å to ~7Å and remained stable especially for the last 200ns), but in absence of cholesterol it increased from ~1Å to ~8Å and did not stabilize even after 900 ns (S4A Fig). This suggests that the relatively higher stability observed within the cholesterol bound MHC-II structures is perhaps due to the cholesterol binding. Similarly, RMSD of the peptide (S4B Fig) remains slightly higher in absence of bound cholesterol. The flexibility of each residue can be inferred from its RMSF values. S5A Fig shows the RMSF for MHC-II chains and domains whereas panel S3B provides the same for peptide in presence and absence of bound cholesterol. The average fluctuations of the residues in both chains of MHC-II were relatively higher for simulation performed without the bound cholesterol (S5A and S6 Figs). Similarly, significantly higher fluctuations within the peptide were also observed for simulation performed without the bound cholesterol (S5B Fig).

### Structural changes within the peptide binding domain in absence of bound cholesterol

**Fig 3:**
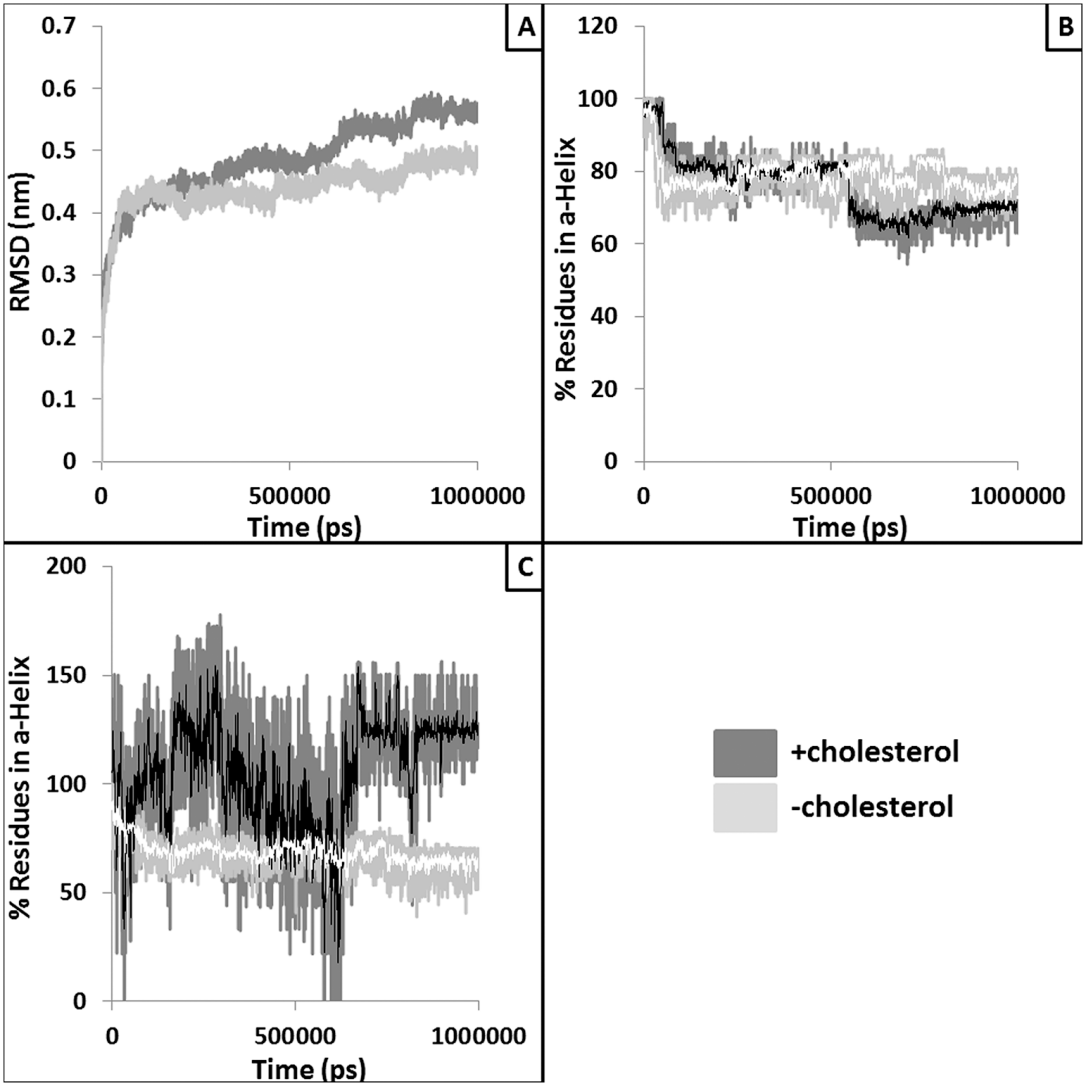
Alteration in overall structure and the helicity of the peptide binding domain (PBD). Panel A provides RMSD trajectories of the PBD extracted from the simulations with (dark grey) and without (light grey) the docked cholesterol. Panel B and C provide the plots where percentages of residues in α-helix conformation are plotted against the simulation time for chain A PBD helix (residues: 50–80) and Chain B PBD helix (residues: 52–89), respectively. White and black lines represent moving averages (period: 10) of the raw data.

**Fig 4:**
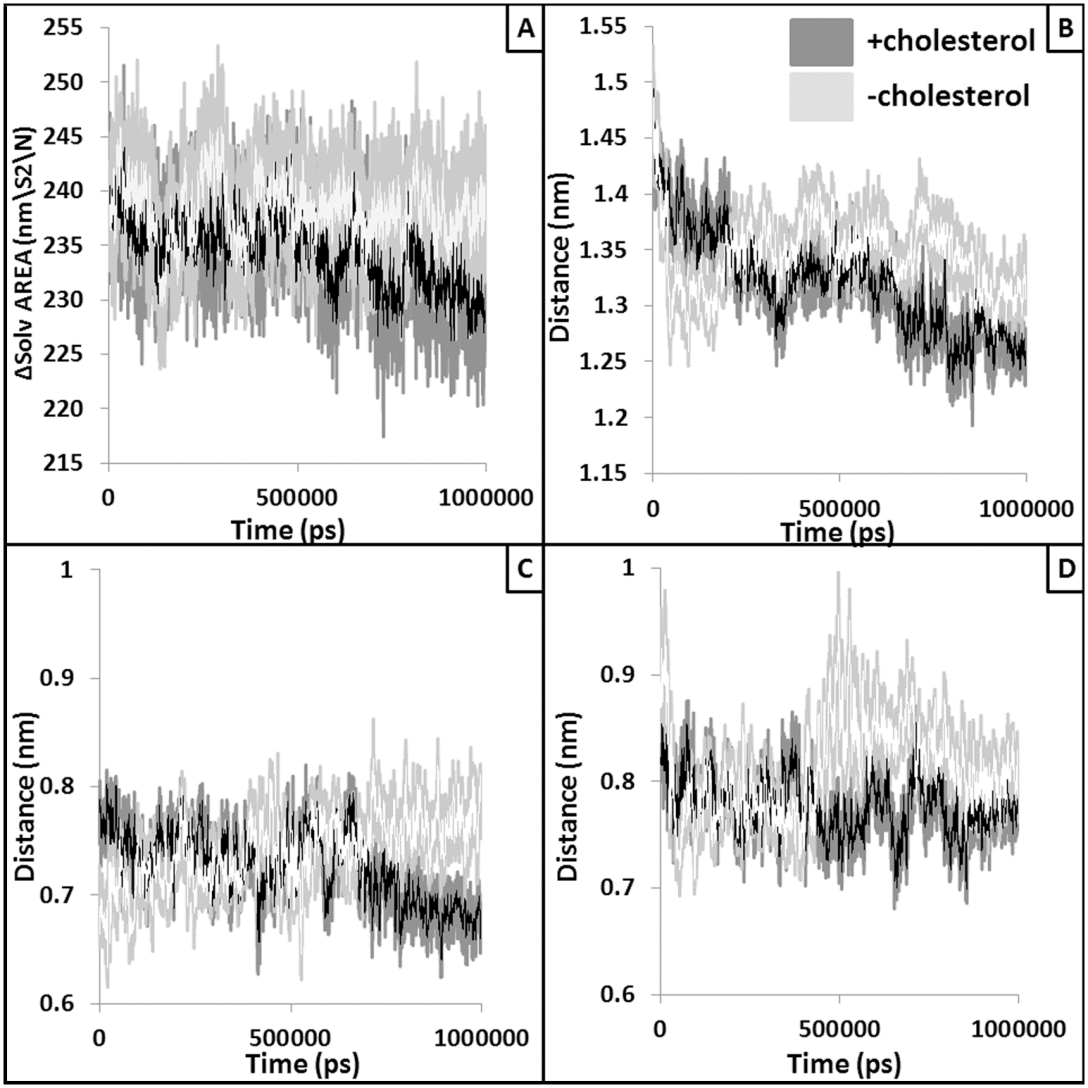
Alteration in the volume and size of the peptide binding pocket. Solvent accessible surface area (SASA) values of the peptide binding region are plotted against the simulation time (panel A). Light grey line represents for without cholesterol runs whereas dark grey line represents SASA values extracted from simulation performed with the docked cholesterol. White and black lines show running average of 10 points. Panel B plots the distance (nm) between two helices of PBD whereas panel C and D shows the average distance between the peptide and the chain A PBD helix and chain B PBD helix, respectively. White and black lines represent moving averages (period: 10) of the raw data.

Peptide binding domain (PBD) of MHC-II is responsible of binding and presenting the antigenic peptide to the T-cell receptor to initiate the immune response. Hence, structural alteration in this domain might lead to loss of peptide binding and/or presentation of peptide to T cells. Structural changes (*e*.*g*., RMSD and percentage helicity) were observed within the PBD helices with respect to presence and absence of the bound cholesterol (Fig 3). Similarly, accessible surface area (ASA) of the PBD was also found to be increased in absence of bound cholesterol (Fig 4A). This could be due to the higher inter-helix and helix-peptide distances observed in absence of bound cholesterol (Fig 4B, 4C and 4D) resulting in reduced peptide binding.

### Structural changes within the middle domain in absence of bound cholesterol

MHC-II middle domain (MID) comprised of immunoglobulin like folds, showed significant difference in fluctuation with respect to presence and absence of bound cholesterol (S5 and S6 Figs). RMSD pattern (S7 Fig) of the domain in presence and absence of cholesterol also showed significance variation. This is interesting as the middle domain usually acts as a connector between TM and the PBD and significant alterations in middle domain conformation and flexibility advocate its role in maintaining the structural stability of the extracellular domains with respect to cholesterol binding at the TM region.

### Structural changes within the TM regions in absence of bound cholesterol

**Fig 5:**
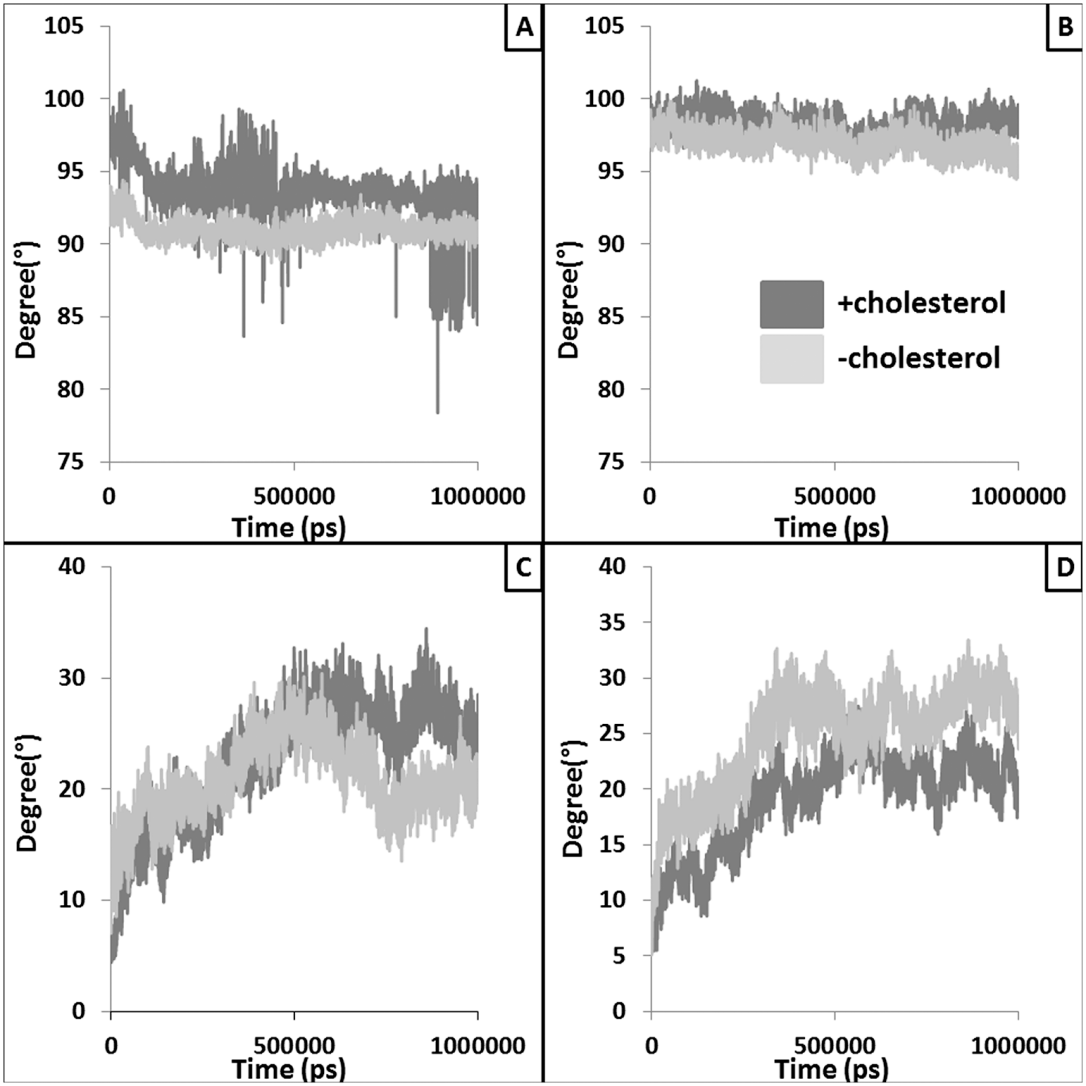
Structural alteration in TM domain with respect to presence and absence of cholesterol. Twist (panel A and B) and tilt (panel C and D) of the TM helices calculated with respect to presence and absence of docked cholesterol are plotted against simulation time. Dark and light grey lines represent average twist and tilt values of TM helices extracted in presence and absence of docked cholesterol.

The tilt and twist of the TM helices are altered markedly with respect to presence and absence of bound cholesterol (Fig 5). Frequencies of residues in helical conformation were calculated from the ensemble TM structures. Similarly, variations in typical intra-helix (i+i4^th^ residue) hydrogen bond length were measured for the TM helices. It is clear from S8A and S8B Fig that chance of a residue to be present in helical conformation is higher for TM region chain A than chain B in presence of bound cholesterol, however, the typical hydrogen bond lengths were not significantly altered (S8C and S8D Fig).

### Cross-correlation of structural fluctuation between MHC-II domains

Striking structural fluctuations were observed within the domains of MHC-II in presence and absence of bound cholesterol. However, it is interesting to investigate whether the fluctuations are correlated between the domains and whether binding of cholesterol impacts these correlations. Hence, cross-correlation or covariance of fluctuation among all residues and domains were calculated and are represented in terms of matrices (S9 Fig) and S1 Table, respectively. S10 Fig suggests that the cholesterol binding probably impacts the cross-correlation between various domains of MHC-II protein as majority of the cases the fold changes of cross-correlation are found to be more than unity in cholesterol bound form compared to the unbound form.

### Energetic changes within the MHC-II in absence of bound cholesterol

**Fig 6:**
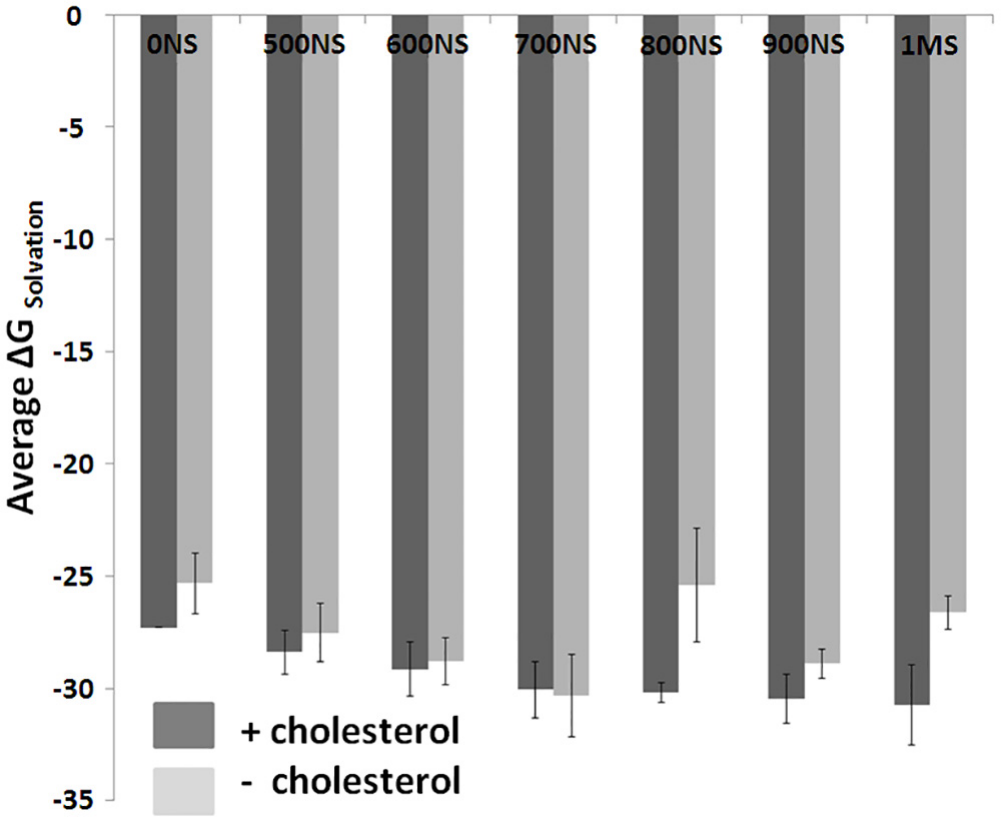
Change in peptide binding energy in PBD with respect to presence and absence of cholesterol. Solvation free energies (Kcal/Mol) of the PBD domain and the bound peptide for a few selected ensemble structures extracted at various time points [from 0NS (nanosecond) to 1MS (microsecond)] are shown. Dark and light grey bars represent complexes extracted from the simulation performed in presence and absence of bound cholesterol, respectively.

As the stability of the MHC-II is closely related to its energetics, the solvation free energies (Kcal/Mol) of the PBD and the bound peptide were calculated using PDBePISA [45] for a few selected ensemble structures extracted at various time points of the simulation and were compared with respect to presence and absence of bound cholesterol. Interestingly, significant reductions in energies were observed for most of the complexes (PBD and peptide) extracted in absence of bound cholesterol (Fig 6). This exercise clearly indicates that the presence of cholesterol indeed impacts the peptide binding energetics at the PBD. However, more detailed and exhaustive study is required for in depth understanding of the energetic changes.

### Assay of kinetic stability using mAB that recognize peptide-MHC complex

**Fig 7:**
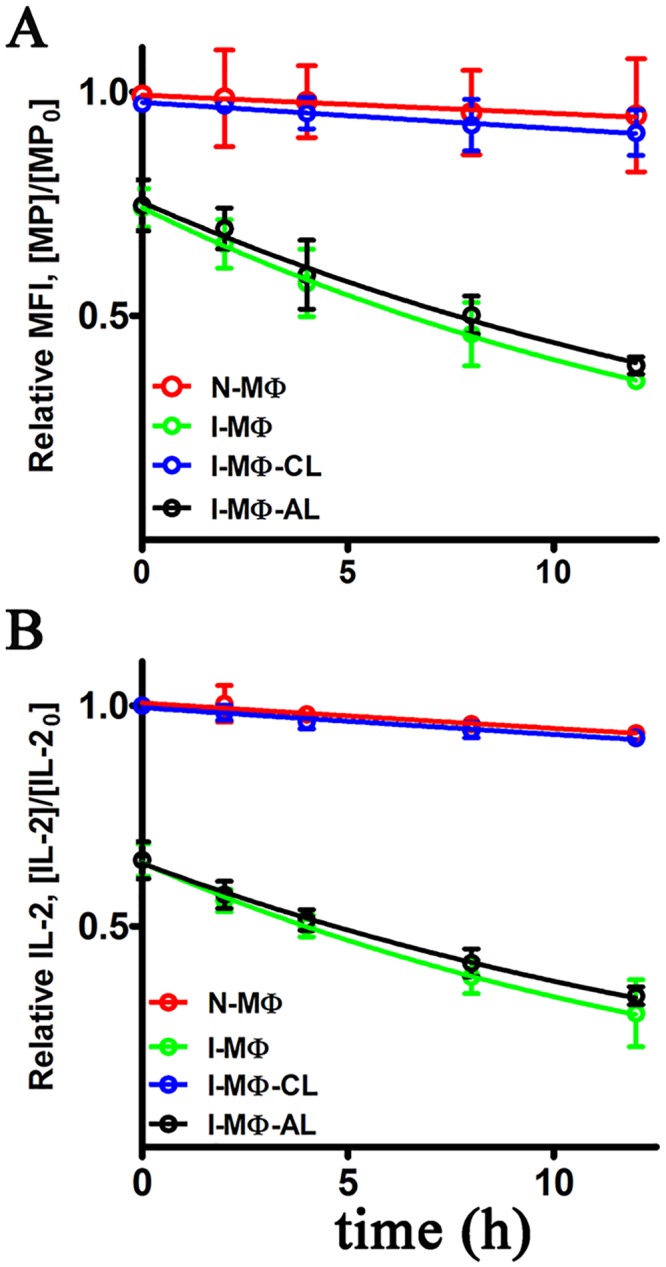
Dissociation of peptide-MHC-II complex on the live cells. N-MΦ (red), I-MΦ (green), I-MΦ-CL (blue) and I-MΦ-AL (black) were pulsed with 20 μM LACK_156-173_ peptide for 6 h at 37°C and this is defined as ‘0’ h time point, followed by washing and then fixed with 4% paraformaldehyde followed by washing and incubated with 50 μM OVA_323-339_. **A,** LACK_156-173_-A^d^ dissociation was measured by FACS. The presence of LACK_156-173_-A^d^ was monitored at 0, 2, 4, 8, and 12 h time points. The cells was stain with m2C44 followed by staining with goat anti-mouse IgG FITC. The data are plotted as the ratio [MP]/[MP_0_] vs time (h). [MP] is MFI at a given time and [MP_0_] is MFI of N-MΦ at 0 h time (time point after 6 h of pulsing). **B,** LACK_156-173_-A^d^ dissociation was monitored by the ability of the complex in the context of appropriate APCs to stimulate T-cells. The presence of LACK_156-173_-A^d^ was monitored by coculturing anti-LACK T cell hybridoma (LMR7.5) with peptide pulsed APCs and the resulting IL-2 production in the supernatant was measured at 0, 2, 4, 8 and 12 h. The data are plotted as the ratio [IL-2]/[IL-2_0_] vs time (h). [IL-2] is the IL-2 produced at a given time and [IL-2_0_] is the IL-2 produced from N-MΦ at 0 h time. The rate of dissociation (k_off_) was calculated by considering 1^st^ order dissociation.

Because of the availability of leishmanial antigen, LACK specific T-cell hybridoma (LMR7.5) of Balb/c origin and mAb that recognize LACK_156-173_-A^d^ (m2C44), the kinetic parameters of peptide-MHC stability was studied with APCs of Balb/c origin. The stability of peptide-MHC complex was monitored by looking at the availability of peptide-MHC complex on the surface and also by their ability to stimulate appropriate T-cells. The availability of LACK_156-173_-A^d^ was monitored using fluorescence labeled antibodies. It was observed that at ‘0’h time point the MFI in I-MΦ was 30% less as compared to N-MΦ. The identical results were observed in the case of I-MΦ-AL. Interestingly, the results were superimposable between N-MΦ and I-MΦ-CL. The rate of dissociation of the peptide-MHC complex in N-MΦ and I-MΦ and I-MΦ-CL and I-MΦ-AL was 1.45μs^-1^, 16.3μs^-1^, 1.68μs^-1^ and 13.4μs^-1^, respectively (Fig 7A).

### Functional assay of kinetic stability of peptide-MHC complex in terms of T-cell activation

We also assessed the availability of LACK_156-173_-A^d^ complex on the cell surfaces of MΦ on the basis of their ability to stimulate anti-LACK T cell hybridoma (LMR7.5). The T cell stimulating ability was measured in terms of IL-2 production. It was observed at ‘0’ h time point the IL-2 production from LMR7.5 was ~ 40% less when I-MΦ or I-MΦ-AL were used as APCs as compared to N-MΦ or I-MΦ-CL. The rate of decreases of IL-2 production in I-MΦ and I-MΦ-AL was 17.1 μs^-1^ and 15.8 μs^-1^ respectively whereas the same for N-MΦ and I-MΦ-CL were 1.29 μs^-1^ and 1.38 μs^-1^ respectively (Fig 7B).

## Discussion

In this investigation we endeavored to learn the ‘complex rules’ of how intracellular LD parasites escape the immune surveillance of the host. The basic tenet of this investigation is based on our past studies where we showed that LD parasites during their intracellular life cycle in MΦ of Balb/c origin take up membrane cholesterol leading to an increase in membrane fluidity [5]. Cholesterol is the major component of eukaryotic bio-membrane and plays a central role in the process of signal transductions [46], maintenance of membrane fluidity [9] and transport [47]. Kala azar patients show dramatic decrease in serum cholesterol as a function of splenic parasite load [48]. Previously, we unveiled the mechanism of hypocholesterolaemia in experimental LD infection and showed that maturation of miR122 in the liver is inhibited via GP63 mediated cleavage of DICER1 [8] and statin induced hypocholesterolaemia show higher organ parasites in experimental infection than the ones without treatment [49]. Here we show that similar to I-MΦ of Balb/c mice [4, 5], I-MΦ of CBA/j mice also showed increased membrane fluidity coupled with defective T-cell stimulating ability and such attributes of I-MΦ can be corrected by liposomal cholesterol but not by liposomal cholesterol analogue or liposomal DPPC. To show that cholesterol mediated effect is specific to I-MΦ, N-MΦ were treated with liposomal cholesterol and such treatment showed hardly any additional effect (Fig 1) indicating that liposomal cholesterol has specific effects on the I-MΦ. Like I-MΦs, LD infected dendritic cell (DC) also showed defective T cell stimulation and treatment with liposomal cholesterol restored T cell stimulating ability (S3 Fig). Liposomal DPPC delivery despite restoring membrane rigidity as shown earlier [50, 51] failed to restore T cell stimulating ability of I-MΦ (Fig 1) suggesting that: a) correction of membrane fluidity of I-MΦ is not enough to stimulate T cell and b) there is a specific need for cholesterol.

Cholesterol’s effects are also possibly due to specific sterol-protein interactions, as shown in the case of a number of membrane bound receptors, such as those for oxytocin, cholecystokinin, galanin and nicotinic acetylcholine [16]. Molecular dynamics (MD) studies revealed that cholesterol physically interacts with nicotinic acetylcholine receptor to prevent the structure from distortion and collapse [52]. Previously, we showed using that transmembrane domain of MHC-II (TM-MHC-II) interacts with cholesterol through CRAC like motifs to bring about conformation change of MHC-II protein [11]. It has been shown through MD studies that cholesterol interacts with rhodopsin to induce local structural perturbation, which in turn brings global conformational rearrangements through rigid body motion [53]. In this study we have investigated how interaction of cholesterol with TM-MHC-II would influence conformation changes in the distal peptide binding groove of the MHC-II protein. The availability of conformation and non-conformation specific anti A^k^ mAb greatly aided in such investigation. The anti A^k^ mAb 11–5.2 recognizes Ia.2 epitope and binds close to the peptide binding groove of MHC-II α chain where arginine-57 and glutamine-75 are critical for binding [20, 21]. Ia.2 is a conformational specific epitope and its expression depends on the presence of specific MHC-II β chain [22]. Recent study from our group [11] and by others [24] showed that conformation change of A^k^ can be monitored by mAb 11–5.2. These studies reinforce that Ia.2 is a conformational epitope and mAb 11–5.2 is a tool to monitor the conformational change in A^k^. We showed that binding of 11–5.2 to I-MΦ was reduced by about 60% as compared to N-MΦ whereas binding of 10–2.16 was comparable between N-MΦ and I-MΦ. This observation indicated that A^k^ molecules are on the surface of I-MΦ, where conformational constrains may limit the binding of 11–5.2 to I-MΦ but not to I-MΦ-CL. This effect was cholesterol specific because the binding of 11–5.2 with I-MΦ was not restored by cholesterol analogue liposome or DPPC liposome treatment.

Here, we also performed exhaustive molecular dynamic simulations to study the effect of cholesterol binding on the TM-MHC-II domain to the distal mid domain and peptide binding groove of MHC-II. Our MD analyses and results suggest marked variations in flexibility and conformational adaptability of the MHC-II protein with respect to cholesterol binding. Our results also provide insight towards the mechanistic details of the probable long range allosteric adaptation observed at MHC-II extracellular domain with respect to binding of cholesterol at the lower base of the TM domain. Local alterations such as tilt and twist of the TM helices vary noticeably with and without the bound cholesterol. Our cross-correlation of flexibility analyses suggest that these changes at the TM-MHC II domain are probably correlated with the conformational variation at the distal extracellular domain. Further, peptide binding energetics was found to be favorable in presence of bound cholesterol indicating stability of peptide-MHC II complex may reduce in absence of cholesterol. To test the prediction that peptide-MHC II stability is compromised in absence of cholesterol we measured peptide-MHC II stability on the cell surface. The stability of peptide-MHC II complex on the cell surface can be measured by two methods: 1) APC was pulsed with peptide followed by fixation (with either glutaraldehyde or paraformaldehyde) and 2) APC was fixed followed by pulsed with peptide [31, 32]. It has been shown that under both experimental conditions stability of peptide-MHC complex is identical therefore fixation of the peptide doesn’t have detrimental effect on the peptide-MHC stability. Here we adopted method 1 and our experimental studies on the kinetic stability of the peptide-MHC complex it was clear that the rate of dissociation of the cognate peptide from the complex was 1.45, 16.3, 13.4 and 1.68 μs^-1^ in N-MΦ, I-MΦ, I-MΦ-AL and I-MΦ-CL, respectively and this was further validated from the progressive decrease in IL-2 production in anti-LACK T-cells as a function of time. This observation shows a clear association between reduced binding of 11–5.2 to cell surface A^k^ and faster dissociation of cognate peptide from peptide-MHC complex in I-MΦ. The above defects were found to be corrected by liposomal cholesterol treatment of I-MΦ. On the basis of our computational data supported by experimental findings, we tend to believe that interactions of cholesterol with TM-MHC-II may allosterically modify the conformation of peptide binding groove and this leads to more stabilization of the peptide-MHC complex on the cell surface under physiological condition than pathological condition. Earlier MD based studies involving serotonin 1_A_ [54, 55] and β2 adrenergic receptors [56] have shown that enrichment of cholesterol within the membrane alters protein oligomerization and stability. Hence, in case of MHC-II, impact of both the bound and membrane enriching cholesterol should be studied to simulate more realistic biological scenario. However, all-atom and/or coarse grain simulations using such systems are out of the scope of this current work and will be addressed in our future studies. The modulation of cholesterol induced conformation change of membrane protein may be extremely complex and have no simple technological (experimental) advances by which it is possible to monitor the nature of changes that alter agonist affinity.

To support the MD study we showed that reduced peptide-MHC-II stability was restored to normal by liposomal cholesterol treatment. Previously we showed that processing of exogenous antigen and also the transport of peptide-MHC-II complex to the cell surface remains unaltered in LD infection [6].

Thus it is conceivable that the conformation of cell surface MHC-II in LD infection may be a key, in part, to understand defective T cell response. There is a report that macrophages from Balb/c mice upon infection with *L*. *donovani* show decrease in B7.1 expression whereas ICAM1 expression is increased. It is not clear whether such effect was due to decrease in membrane cholesterol. Thus all the membrane proteins are not equally affected in *L*. *donovani* infection [57]. We also showed that in infected macrophages there is an increase in lateral mobility of membrane protein [4] and such macrophages are incapable to form effective immunological synapse with antigen specific T-cells [5]. Our study supported by a previous observation by others in *Leishmania major* infection where it was shown that under heavily parasitized condition the infected dendritic cells lack peptide-MHC-II (LACK_156-173_-A^d^) complex [30]. The present study is bringing a mechanistic view to explain how decrease in membrane cholesterol in I-MΦ may influence conformation of membrane associated protein like MHC-II in this case. It may be recalled in this context that liposomal cholesterol shows dramatic therapeutic effect in experimental visceral leishmaniasis [5]. Beneficial role of cholesterol has been noted from a number of epidemiological studies [58] and clinical studies in pulmonary tuberculosis patients [59]. Patients with Smith-Lemli-Opitz syndrome who were treated with a pure cholesterol suspension orally also showed improvement in symptoms associated with the disease [60].

In summary, it appears that LD parasites by decreasing membrane cholesterol during their intracellular life cycle may have altered the conformation of MHC-II molecules with direct bearing on the compromised agonist affinity leading to faster dissociation of cognate peptide from the peptide-MHC-II complex which could be corrected by liposomal cholesterol delivery.

## Supporting Information

S1 Fig Infection macrophages with LD. Stationary phase promastigotes were used for *in vitro* infection of MΦs. The MΦs after 48 h incubation in complete medium, were challenged with LD promastigotes (macrophage to parasite ratio 1:10) and incubated further for 6 h at 37°C. Excess parasites were then washed off with serum free medium. The intracellular parasites were enumerated microscopically at 6 h and 48 h after washing. The results were expressed as the number of parasites/cell.
(TIF)

S2 Fig Three-dimensional (3D) model of MHC-II dimer along with the docked cholesterol moieties. MHC-II molecule is embedded in POPC bilayer where chain A (red), chain B (cyan), cholesterol (green) and peptide (yellow) are marked in different colors.
(TIF)

S3 Fig Fold change of cross-correlation of fluctuation between MHC-II domains with respect to presence and absence of cholesterol. N-DC, I-DC, I-DC-CL, I-DC-AL, I-DC-DPPC and N-DC-CL were cocultured with anti-LACK T cell hybridoma (LMR7.5) in presence of 5 μM LACK_156-173_ peptide. The resulting IL-2 production was measured by ELISA.
(TIF)

S4 Fig Alteration in overall structure with respect to presence and absence of cholesterol. Time evolution of root mean square deviation (RMSD) trajectory analysis of the MHC-II and the bound peptide. Dark and light grey lines represent average RMSDs for MHC-II with and without the docked cholesterol, respectively. Panel A provides RMSD trajectories for MHC-II and the peptide whereas panel B shows trajectory for the peptide only.
(TIF)

S5 Fig Alteration in residue fluctuation pattern with respect to presence and absence of cholesterol. Root mean square fluctuations (RMSF) of MHC-II (panel A: chain A, chain B) and peptide (panel B) residues were plotted with respect to the simulation time. Light grey lines represent average RMSF for without cholesterol simulations while dark grey lines represent average RMSF for with cholesterol runs.
(TIF)

S6 Fig Alteration in domain fluctuation pattern with respect to presence and absence of cholesterol. Average RMSF of peptide binding domain (PBD), middle domain (MID) and transmembrane domain (TM) are plotted with and without the docked cholesterols. PBD_A: Peptide Binding Domain of Chain A; PBD_B: Peptide Binding Domain of Chain B; MID_A: Middle Domain of Chain A; MID_B: Middle Domain of Chain B; TM_A: Transmembrane Domain of Chain A; TM_B: Transmembrane Domain of Chain B. ** denotes where p value << 0.001.
(TIF)

S7 Fig RMSD of middle domain. Dark grey and light grey lines represent average RMSD of MHC-II middle domain extracted from simulations performed with and without the docked cholesterol.
(TIF)

S8 Fig Alteration in helix properties of the TM helices. Frequencies of individual residues located in α-helical conformation for chain A (panel A) and chain B (panel B) TM domains are plotted against simulation time. Panel C and D plot the inter residue distances (of *i* and *i+4th* residue) within the chain A (panel C) and chain B (panel D) TM helices. White and black lines represent moving averages (period: 10) of the raw data.
(TIF)

S9 Fig Covariance of residue fluctuation. Covariance or cross-correlation of fluctuation between any pair of residues was calculated where higher correlation coefficient reflects higher covariance. All-to-all matrices of the correlation coefficients are provided for MHC-II residues when simulated with cholesterol (panel A, B, C representing correlation from three individual simulation runs) and without cholesterol (panel D, E, F).
(TIF)

S10 Fig Fold change of cross-correlation of fluctuation between MHC-II domains with respect to presence and absence of cholesterol. Fold change of cross-correlation of fluctuation (FC_ACR_ = ACR_+CHL_ / ACR_-CHL_) between various domains (Panel A: PBD domain and TM domain; Panel B: MID domain and TM domain; Panel C: PBD domain and MID domain) of MHC-II are plotted where *FC*_*ACR*_ is fold change of average cross-correlation and *ACR*_*+CHL*_, *ACR*_*-CHL*_ represent average cross-relation between domains (as shown in S1G and S1H Table) in presence and absence of cholesterol, respectively.
(TIF)

S1 Table Cross-correlation of fluctuation for MHC-II domains and peptide in absence (A-C), presence (D-F) of docked cholesterol and their average (G and H) for three simulation runs. (A-C) Cross-correlation of fluctuation in absence of cholesterol for simulation RUN1, RUN2 and RUN3, respectively. (D-F) Cross-correlation of fluctuation in presence of cholesterol for simulation RUN1, RUN2 and RUN3, respectively. (G) Average correlation coefficient values of fluctuation in absence of cholesterol for simulation RUN1, RUN2 and RUN3. (H) Average correlation coefficient values of fluctuation in presence of cholesterol for simulation RUN1, RUN2 and RUN3. PBD_A: Peptide Binding Domain of Chain A; PBD_B: Peptide Binding Domain of Chain B; MID_A: Middle Domain of Chain A; MID_B: Middle Domain of Chain B; TM_A: Transmembrane Domain of Chain A; TM_B: Transmembrane Domain of Chain B.
(DOCX)
