# Supplementary figures and images for: Cholesterol Corrects Altered Conformation of MHC-II Protein in Leishmania donovani Infected Macrophages: Implication in Therapy
Source: PLoS Negl Trop Dis. 2016 May 23;10(5):e0004710. doi: 10.1371/journal.pntd.0004710 (PMC4877013; doi:10.1371/journal.pntd.0004710)

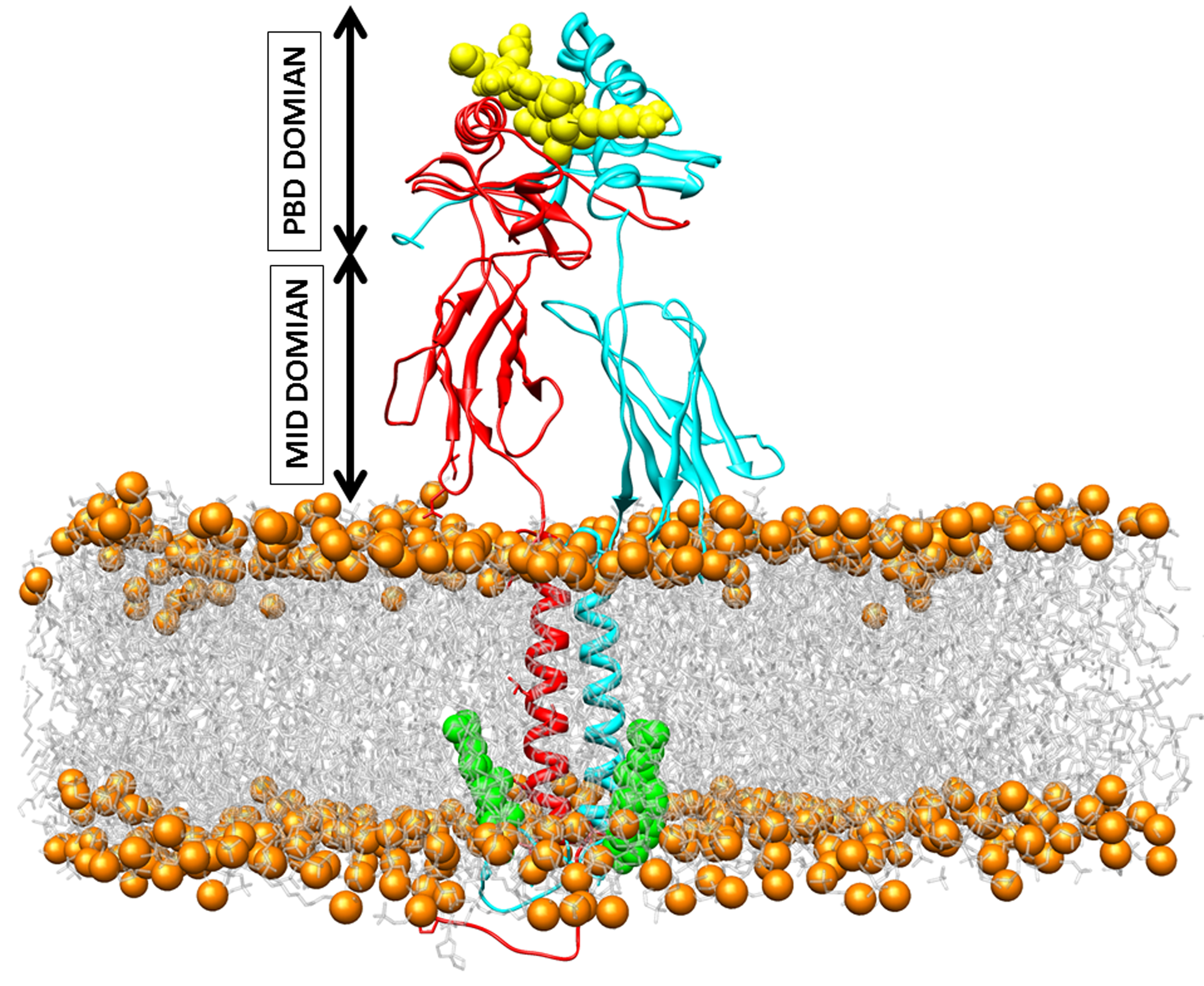

Supplement: S2 Fig — MHC-II molecule is embedded in POPC bilayer where chain A (red), chain B (cyan), cholesterol (green) and peptide (yellow) are marked in different colors. (TIF) [file pntd.0004710.s002.tif]

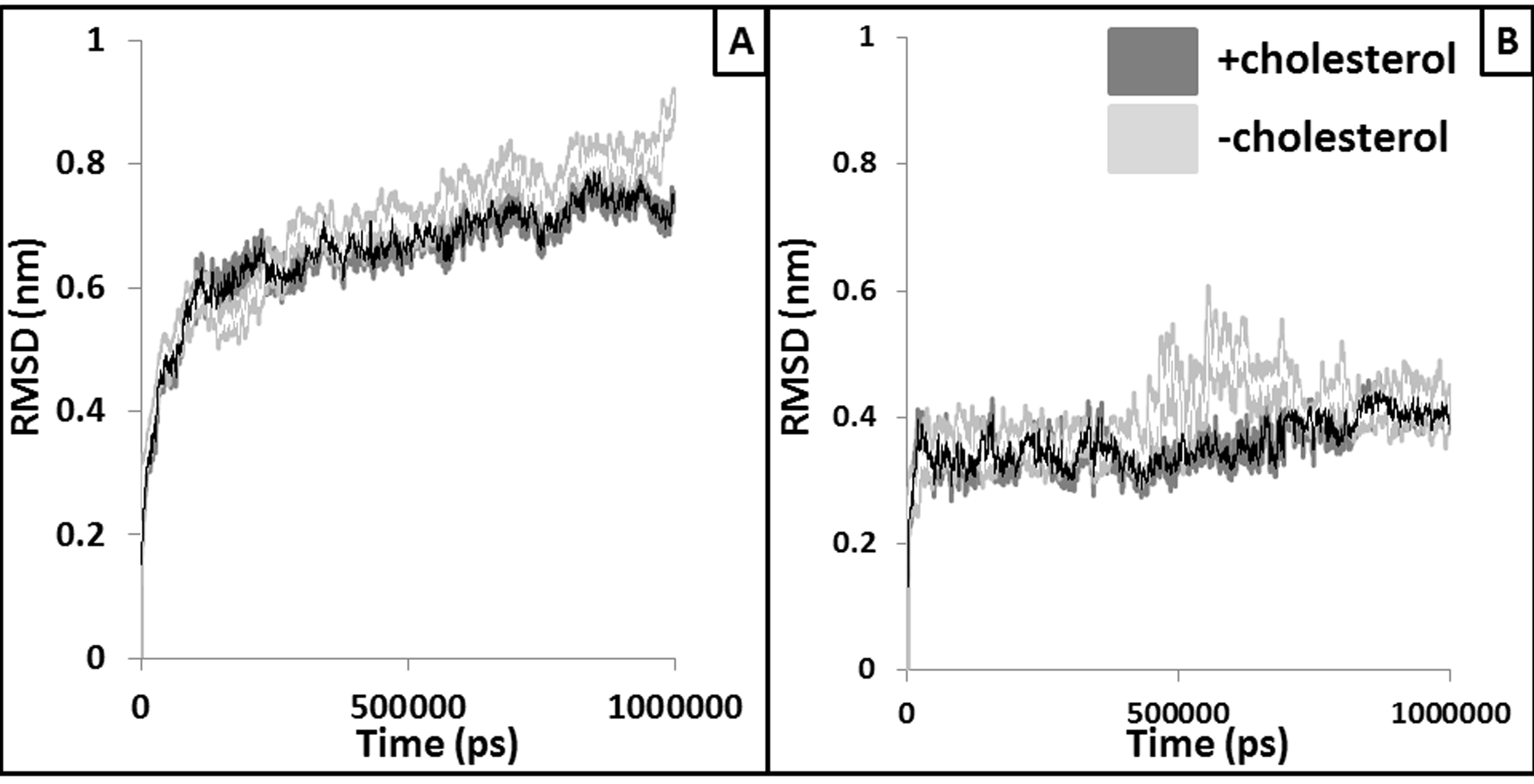

Supplement: S4 Fig — Time evolution of root mean square deviation (RMSD) trajectory analysis of the MHC-II and the bound peptide. Dark and light grey lines represent average RMSDs for MHC-II with and without the docked cholesterol, respectively. Panel A provides RMSD trajectories for MHC-II and the peptide whereas panel B shows trajectory for the peptide only. (TIF) [file pntd.0004710.s004.tif]

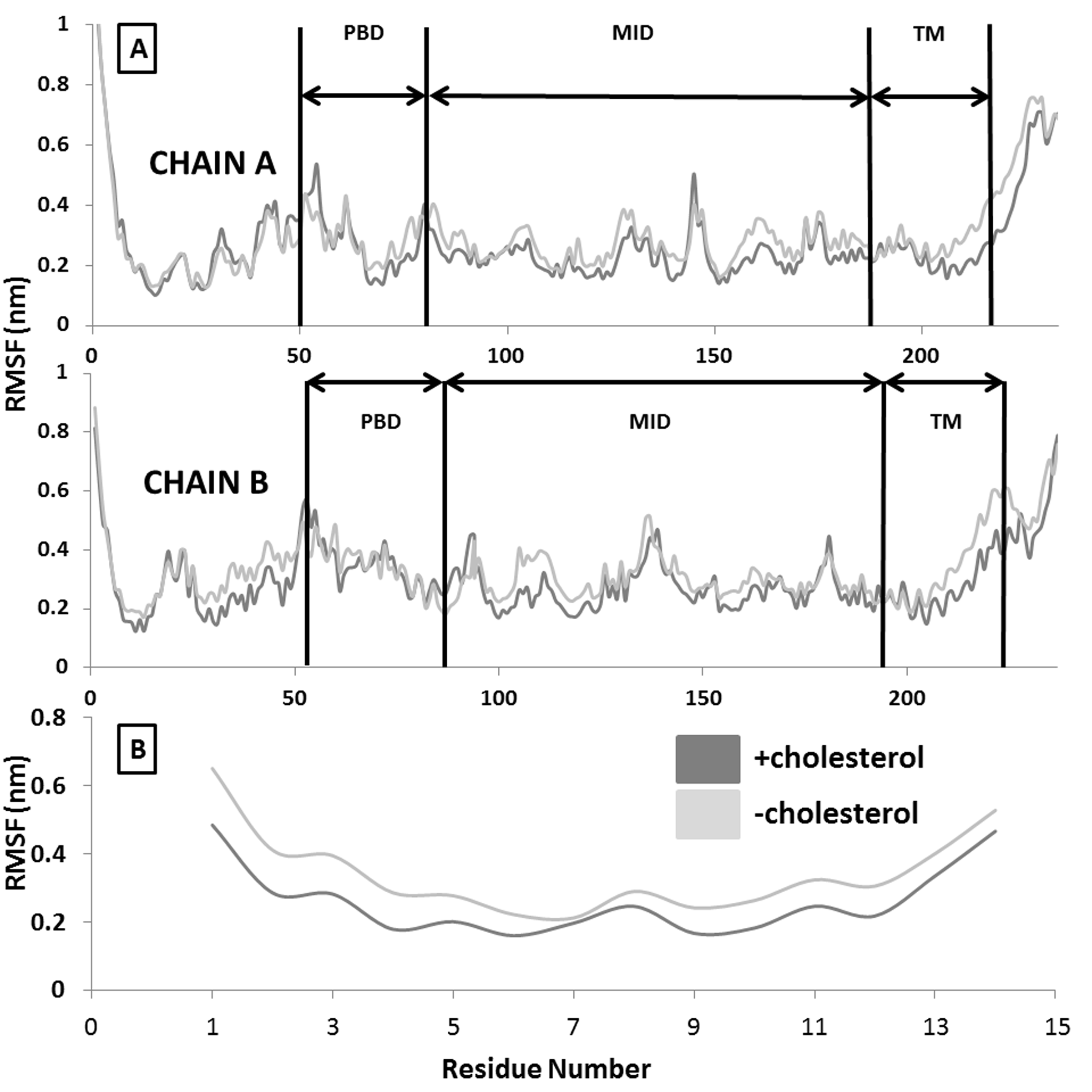

Supplement: S5 Fig — Root mean square fluctuations (RMSF) of MHC-II (panel A: chain A, chain B) and peptide (panel B) residues were plotted with respect to the simulation time. Light grey lines represent average RMSF for without cholesterol simulations while dark grey lines represent average RMSF for with cholesterol runs. (TIF) [file pntd.0004710.s005.tif]

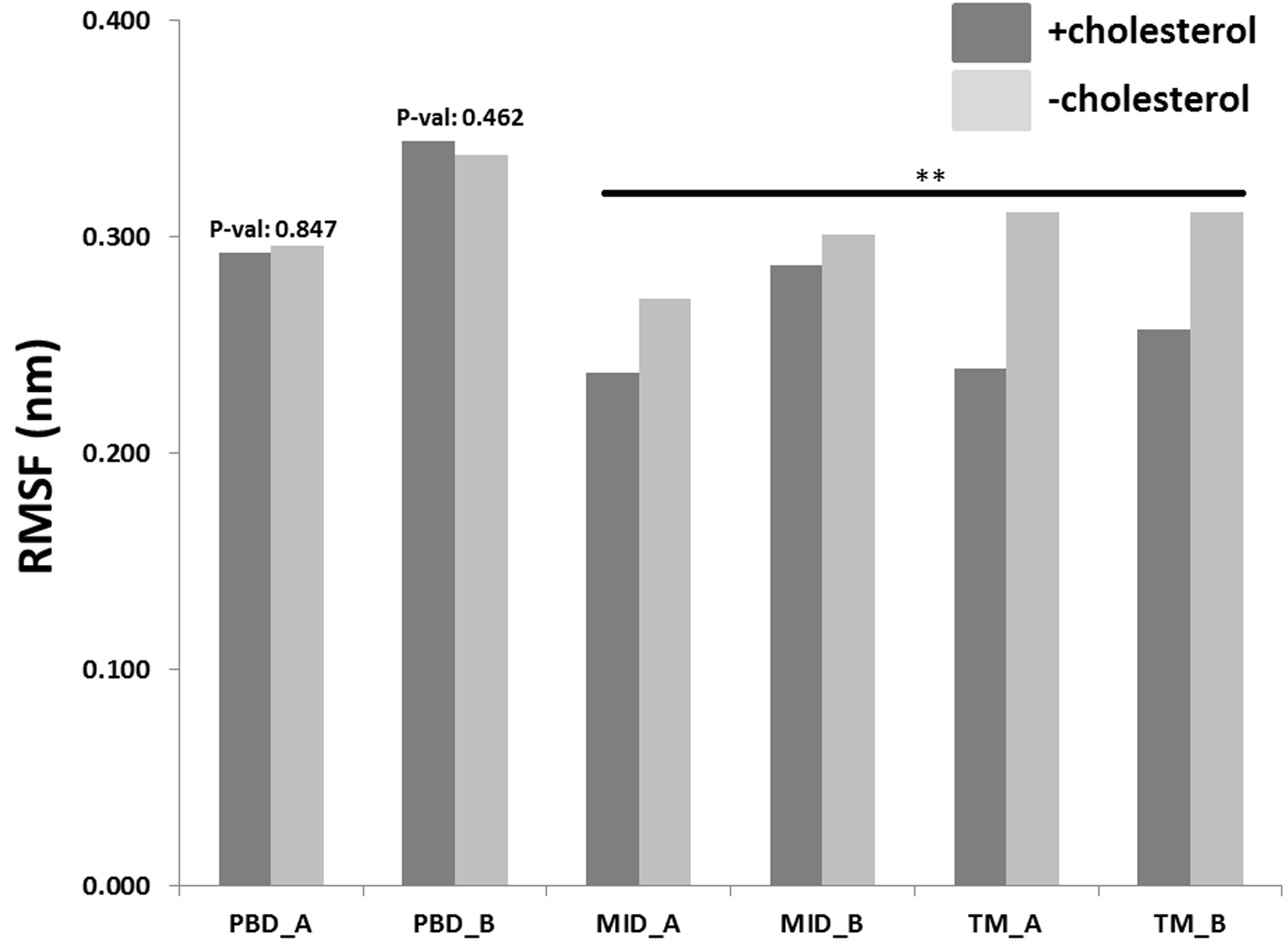

Supplement: S6 Fig — Average RMSF of peptide binding domain (PBD), middle domain (MID) and transmembrane domain (TM) are plotted with and without the docked cholesterols. PBD_A: Peptide Binding Domain of Chain A; PBD_B: Peptide Binding Domain of Chain B; MID_A: Middle Domain of Chain A; MID_B: Middle Domain of Chain B; TM_A: Transmembrane Domain of Chain A; TM_B: Transmembrane Domain of Chain B. ** denotes where p value << 0.001. (TIF) [file pntd.0004710.s006.tif]

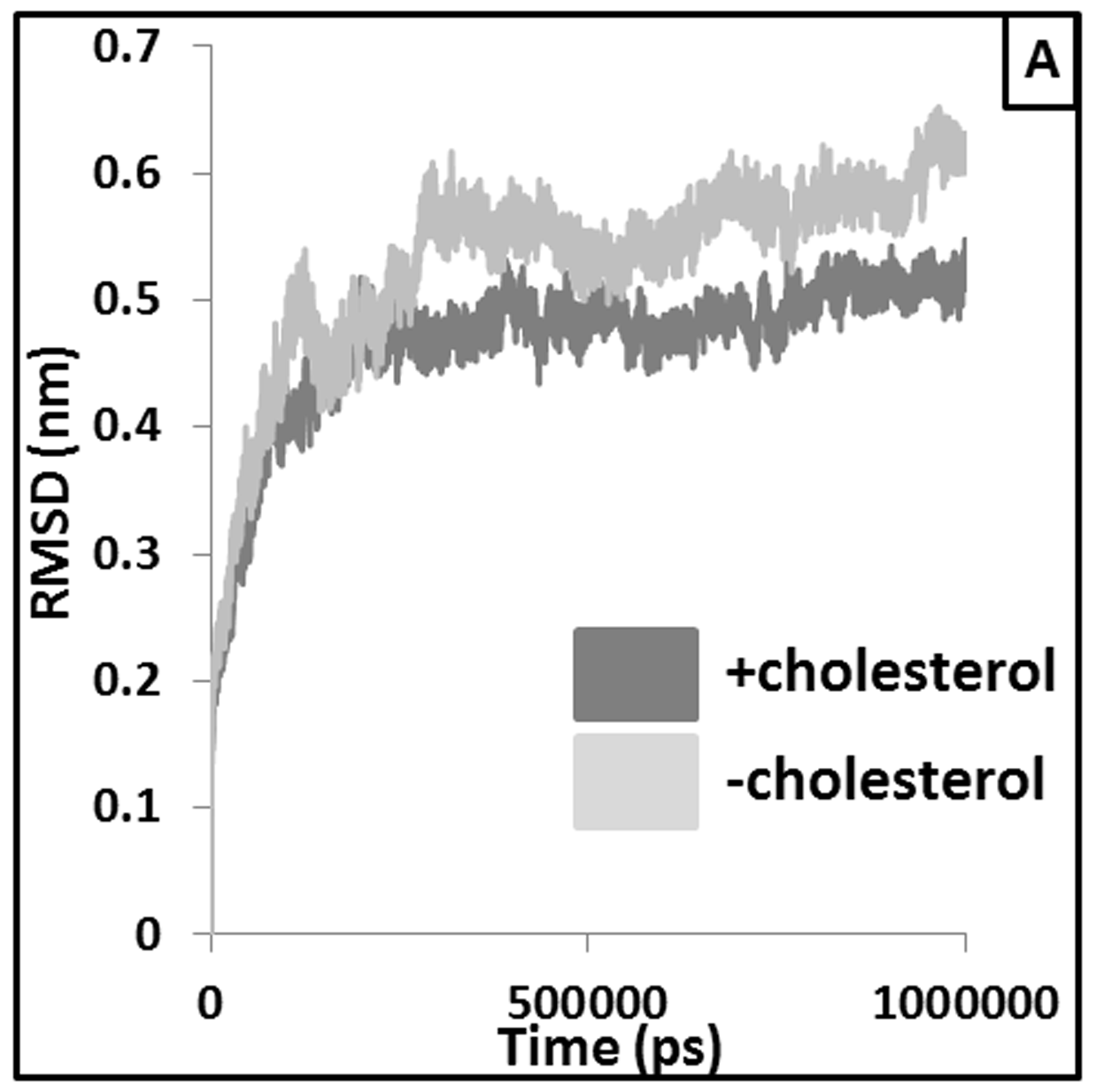

Supplement: S7 Fig — Dark grey and light grey lines represent average RMSD of MHC-II middle domain extracted from simulations performed with and without the docked cholesterol. (TIF) [file pntd.0004710.s007.tif]

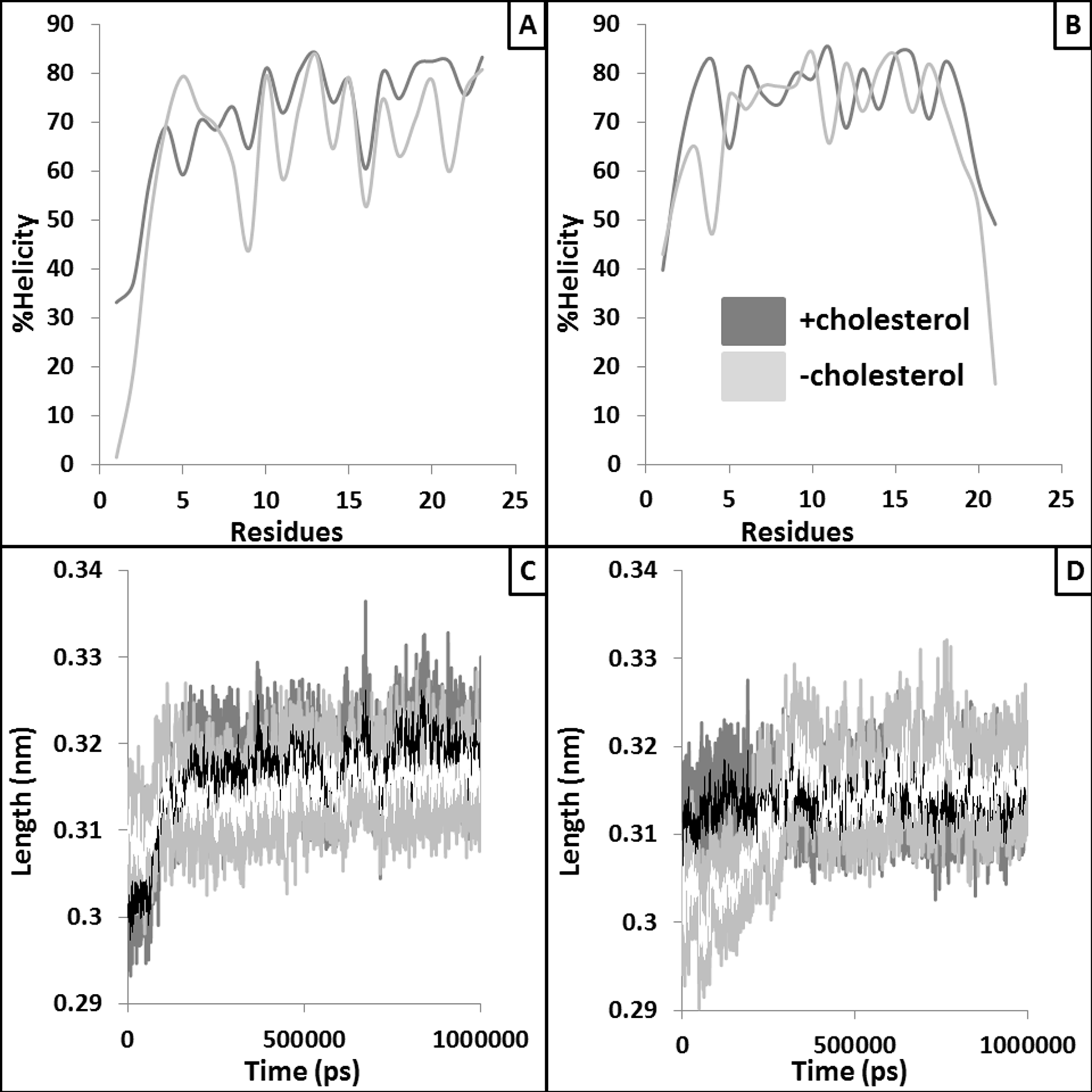

Supplement: S8 Fig — Frequencies of individual residues located in α-helical conformation for chain A (panel A) and chain B (panel B) TM domains are plotted against simulation time. Panel C and D plot the inter residue distances (of i and i+4th residue) within the chain A (panel C) and chain B (panel D) TM helices. White and black lines represent moving averages (period: 10) of the raw data. (TIF) [file pntd.0004710.s008.tif]

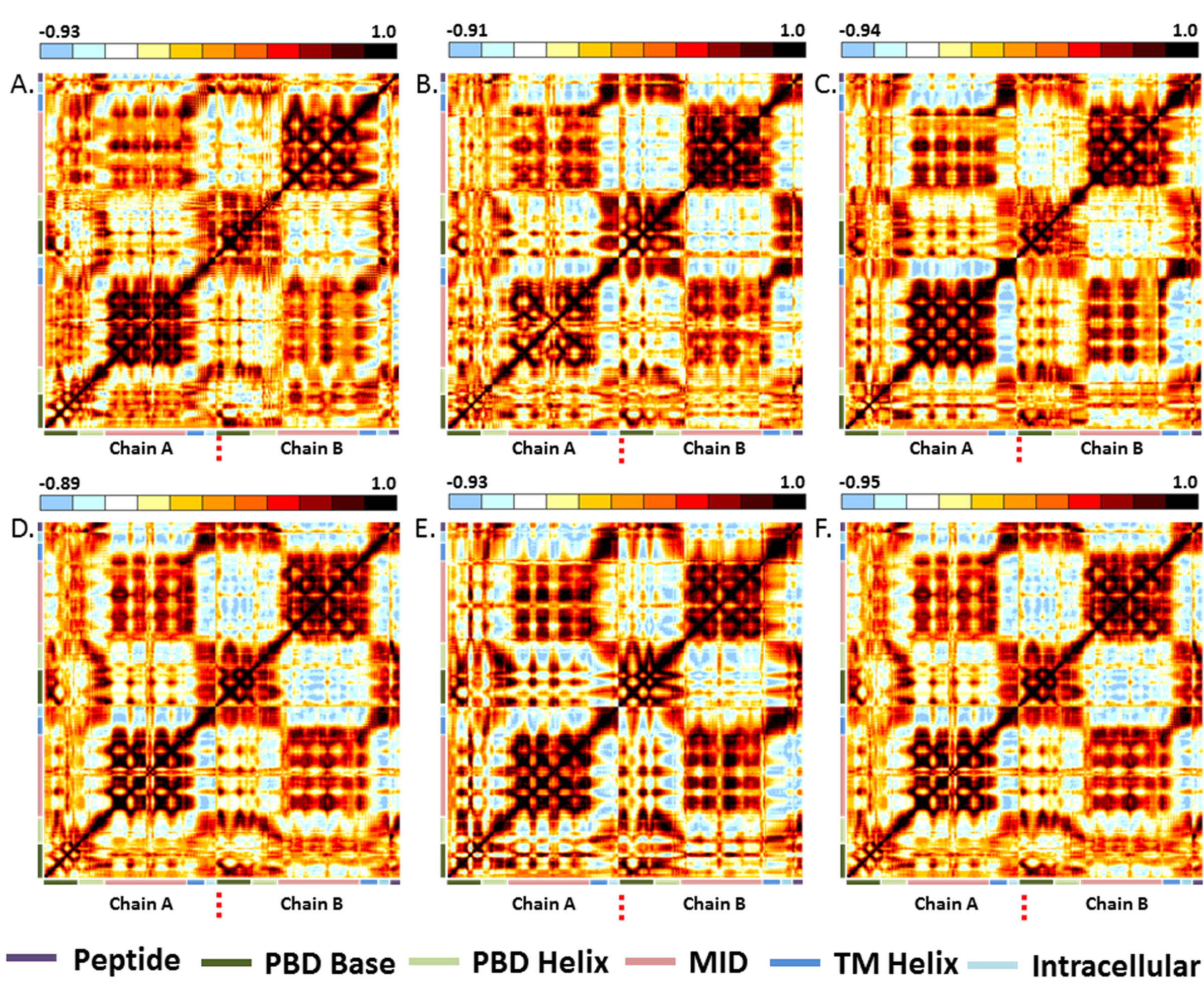

Supplement: S9 Fig — Covariance or cross-correlation of fluctuation between any pair of residues was calculated where higher correlation coefficient reflects higher covariance. All-to-all matrices of the correlation coefficients are provided for MHC-II residues when simulated with cholesterol (panel A, B, C representing correlation from three individual simulation runs) and without cholesterol (panel D, E, F). (TIF) [file pntd.0004710.s009.tif]

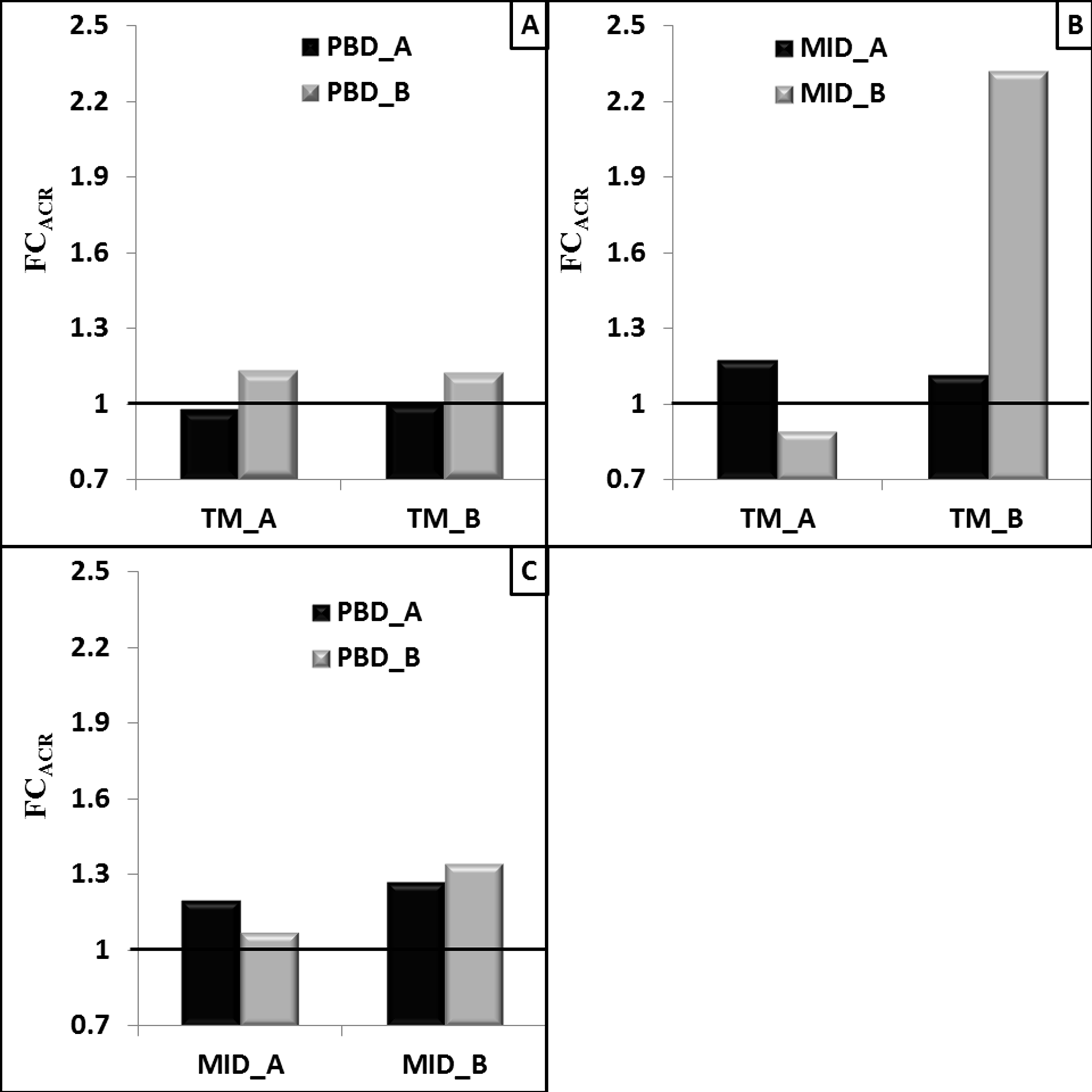

Supplement: S10 Fig — Fold change of cross-correlation of fluctuation (FCACR = ACR+CHL / ACR-CHL) between various domains (Panel A: PBD domain and TM domain; Panel B: MID domain and TM domain; Panel C: PBD domain and MID domain) of MHC-II are plotted where FCACR is fold change of average cross-correlation and ACR+CHL, ACR-CHL represent average cross-relation between domains (as shown in S1G and S1H Table) in presence and absence of cholesterol, respectively. (TIF) [file pntd.0004710.s010.tif]
