# Supplementary material for: Cholesterol Corrects Altered Conformation of MHC-II Protein in Leishmania donovani Infected Macrophages: Implication in Therapy
Source: PLoS Negl Trop Dis. 2016 May 23;10(5):e0004710. doi: 10.1371/journal.pntd.0004710 (PMC4877013; doi:10.1371/journal.pntd.0004710)
Supplement: S1 Table — (A-C) Cross-correlation of fluctuation in absence of cholesterol for simulation RUN1, RUN2 and RUN3, respectively. (D-F) Cross-correlation of fluctuation in presence of cholesterol for simulation RUN1, RUN2 and RUN3, respectively. (G) Average correlation coefficient values of fluctuation in absence of cholesterol for simulation RUN1, RUN2 and RUN3. (H) Average correlation coefficient values of fluctuation in presence of cholesterol for simulation RUN1, RUN2 and RUN3. PBD_A: Peptide Binding Domain of Chain A; PBD_B: Peptide Binding Domain of Chain B; MID_A: Middle Domain of Chain A; MID_B: Middle Domain of Chain B; TM_A: Transmembrane Domain of Chain A; TM_B: Transmembrane Domain of Chain B. (DOCX) [file pntd.0004710.s011.docx]

**Table SI: Cross-correlation of fluctuation for MHC-II domains and peptide in absence (A-C), presence (D-F) of docked cholesterol and their average (G and H) for three simulation runs.**

| **A.** | **PBD_A** | **MID_A** | **TM_A** | **PBD_B** | **MID_B** | **TM_B** | **Peptide** | **B.** | **PBD_A** | **MID_A** | **TM_A** | **PBD_B** | **MID_B** | **TM_B** | **Peptide** | **C.** | **PBD_A** | **MID_A** | **TM_A** | **PBD_B** | **MID_B** | **TM_B** | **Peptide** |
| --- | --- | --- | --- | --- | --- | --- | --- | --- | --- | --- | --- | --- | --- | --- | --- | --- | --- | --- | --- | --- | --- | --- | --- |
| **PBD_A** | **0.36** | **-0.28** | **0.01** | **0.22** | **-0.22** | **-0.03** | **0.36** | **PBD_A** | **0.35** | **-0.28** | **-0.04** | **0.30** | **-0.32** | **0.05** | **0.41** | **PBD_A** | **0.48** | **-0.45** | **0.03** | **0.49** | **-0.47** | **0.00** | **0.55** |
| **MID_A** | **-0.28** | **0.47** | **-0.19** | **-0.32** | **0.23** | **-0.10** | **-0.41** | **MID_A** | **-0.28** | **0.57** | **-0.32** | **-0.32** | **0.32** | **-0.40** | **-0.51** | **MID_A** | **-0.45** | **0.67** | **-0.30** | **-0.45** | **0.45** | **-0.22** | **-0.54** |
| **TM_A** | **0.01** | **-0.19** | **0.33** | **0.09** | **-0.03** | **0.21** | **0.11** | **TM_A** | **-0.04** | **-0.32** | **0.62** | **-0.13** | **0.10** | **0.63** | **0.06** | **TM_A** | **0.03** | **-0.30** | **0.61** | **-0.17** | **0.01** | **0.59** | **0.01** |
| **PBD_B** | **0.22** | **-0.32** | **0.09** | **0.43** | **-0.37** | **-0.10** | **0.44** | **PBD_B** | **0.30** | **-0.32** | **-0.13** | **0.49** | **-0.48** | **-0.09** | **0.49** | **PBD_B** | **0.49** | **-0.45** | **-0.17** | **0.81** | **-0.68** | **-0.26** | **0.68** |
| **MID_B** | **-0.22** | **0.23** | **-0.03** | **-0.37** | **0.56** | **0.13** | **-0.42** | **MID_B** | **-0.32** | **0.32** | **0.10** | **-0.48** | **0.59** | **0.07** | **-0.52** | **MID_B** | **-0.47** | **0.45** | **0.01** | **-0.68** | **0.72** | **0.11** | **-0.62** |
| **TM_B** | **-0.03** | **-0.10** | **0.21** | **-0.10** | **0.13** | **0.39** | **-0.03** | **TM_B** | **0.05** | **-0.40** | **0.63** | **-0.09** | **0.07** | **0.73** | **0.14** | **TM_B** | **0.00** | **-0.22** | **0.59** | **-0.26** | **0.11** | **0.63** | **-0.06** |
| **Peptide** | **0.36** | **-0.41** | **0.11** | **0.44** | **-0.42** | **-0.03** | **0.58** | **Peptide** | **0.41** | **-0.51** | **0.06** | **0.49** | **-0.52** | **0.14** | **0.65** | **Peptide** | **0.55** | **-0.54** | **0.01** | **0.68** | **-0.62** | **-0.06** | **0.70** |
| **D.** | **PBD_A** | **MID_A** | **TM_A** | **PBD_B** | **MID_B** | **TM_B** | **Peptide** | **E.** | **PBD_A** | **MID_A** | **TM_A** | **PBD_B** | **MID_B** | **TM_B** | **Peptide** | **F.** | **PBD_A** | **MID_A** | **TM_A** | **PBD_B** | **MID_B** | **TM_B** | **Peptide** |
| **PBD_A** | **0.42** | **-0.23** | **0.03** | **0.04** | **-0.09** | **0.04** | **0.31** | **PBD_A** | **0.29** | **-0.09** | **-0.14** | **0.09** | **-0.09** | **-0.04** | **0.25** | **PBD_A** | **0.37** | **-0.30** | **0.04** | **0.24** | **-0.29** | **0.03** | **0.36** |
| **MID_A** | **-0.23** | **0.57** | **-0.07** | **-0.34** | **0.14** | **-0.06** | **-0.39** | **MID_A** | **-0.09** | **0.41** | **-0.14** | **-0.34** | **0.22** | **-0.08** | **-0.33** | **MID_A** | **-0.30** | **0.60** | **-0.22** | **-0.28** | **0.19** | **-0.31** | **-0.44** |
| **TM_A** | **0.03** | **-0.07** | **0.40** | **0.07** | **-0.13** | **0.33** | **0.00** | **TM_A** | **-0.14** | **-0.14** | **0.41** | **0.06** | **-0.11** | **0.27** | **0.02** | **TM_A** | **0.04** | **-0.22** | **0.31** | **0.04** | **0.00** | **0.31** | **0.08** |
| **PBD_B** | **0.04** | **-0.34** | **0.07** | **0.38** | **-0.28** | **-0.05** | **0.23** | **PBD_B** | **0.09** | **-0.34** | **0.06** | **0.50** | **-0.43** | **-0.09** | **0.41** | **PBD_B** | **0.24** | **-0.28** | **0.04** | **0.32** | **-0.32** | **0.00** | **0.37** |
| **MID_B** | **-0.09** | **0.14** | **-0.13** | **-0.28** | **0.52** | **0.14** | **-0.21** | **MID_B** | **-0.09** | **0.22** | **-0.11** | **-0.43** | **0.60** | **0.05** | **-0.36** | **MID_B** | **-0.29** | **0.19** | **0.00** | **-0.32** | **0.50** | **0.16** | **-0.38** |
| **TM_B** | **0.04** | **-0.06** | **0.33** | **-0.05** | **0.14** | **0.74** | **-0.15** | **TM_B** | **-0.04** | **-0.08** | **0.27** | **-0.09** | **0.05** | **0.38** | **0.01** | **TM_B** | **0.03** | **-0.31** | **0.31** | **0.00** | **0.16** | **0.50** | **0.06** |
| **Peptide** | **0.31** | **-0.39** | **0.00** | **0.23** | **-0.21** | **-0.15** | **0.49** | **Peptide** | **0.25** | **-0.33** | **0.02** | **0.41** | **-0.36** | **0.01** | **0.53** | **Peptide** | **0.36** | **-0.44** | **0.08** | **0.37** | **-0.38** | **0.06** | **0.54** |
| **G.** | **PBD_A** | **MID_A** | **TM_A** | **PBD_B** | **MID_B** | **TM_B** | **Peptide** | **H.** | **PBD_A** | **MID_A** | **TM_A** | **PBD_B** | **MID_B** | **TM_B** | **Peptide** |  | | | | | | | |
| **PBD_A** | **0.40** | **-0.34** | **0.00** | **0.34** | **-0.34** | **0.01** | **0.44** | **PBD_A** | **0.36** | **-0.21** | **-0.02** | **0.12** | **-0.16** | **0.01** | **0.31** |  |  |  |  |  |  |  |  |
| **MID_A** | **-0.34** | **0.57** | **-0.27** | **-0.36** | **0.33** | **-0.24** | **-0.49** | **MID_A** | **-0.21** | **0.53** | **-0.14** | **-0.32** | **0.18** | **-0.15** | **-0.38** |  |  |  |  |  |  |  |  |
| **TM_A** | **0.00** | **-0.27** | **0.52** | **-0.07** | **0.03** | **0.48** | **0.06** | **TM_A** | **-0.02** | **-0.14** | **0.37** | **0.05** | **-0.08** | **0.30** | **0.04** |  |  |  |  |  |  |  |  |
| **PBD_B** | **0.34** | **-0.36** | **-0.07** | **0.58** | **-0.51** | **-0.15** | **0.53** | **PBD_B** | **0.12** | **-0.32** | **0.05** | **0.40** | **-0.34** | **-0.05** | **0.34** |  |  |  |  |  |  |  |  |
| **MID_B** | **-0.34** | **0.33** | **0.03** | **-0.51** | **0.63** | **0.10** | **-0.52** | **MID_B** | **-0.16** | **0.18** | **-0.08** | **-0.34** | **0.54** | **0.12** | **-0.32** |  |  |  |  |  |  |  |  |
| **TM_B** | **0.01** | **-0.24** | **0.48** | **-0.15** | **0.10** | **0.58** | **0.02** | **TM_B** | **0.01** | **-0.15** | **0.30** | **-0.05** | **0.12** | **0.54** | **-0.03** |  |  |  |  |  |  |  |  |
| **Peptide** | **0.44** | **-0.49** | **0.06** | **0.53** | **-0.52** | **0.02** | **0.64** | **Peptide** | **0.31** | **-0.38** | **0.04** | **0.34** | **-0.32** | **-0.03** | **0.52** |  |  |  |  |  |  |  |  |

**(A**-**C**) Cross-correlation of fluctuation in absence of cholesterol for simulation RUN1, RUN2 and RUN3 respectively. **(D**-**F**) Cross-correlation of fluctuation in presence of cholesterol for simulation RUN1, RUN2 and RUN3 respectively. (**G**) Average correlation coefficient values of fluctuation in absence of cholesterol for simulation RUN1, RUN2 and RUN3. (**H**) Average correlation coefficient values of fluctuation in presence of cholesterol for simulation RUN1, RUN2 and RUN3. PBD_A: Peptide Binding Domain of Chain A; PBD_B: Peptide Binding Domain of Chain B; MID_A: Middle Domain of Chain A; MID_B: Middle Domain of Chain B; TM_A: Transmembrane Domain of Chain A; TM_B: Transmembrane Domain of Chain B.
